# The different natural estrogens promote endothelial healing through distinct cell targets

**DOI:** 10.1172/jci.insight.161284

**Published:** 2023-02-02

**Authors:** Morgane Davezac, Rana Zahreddine, Melissa Buscato, Natalia F. Smirnova, Chanaelle Febrissy, Henrik Laurell, Silveric Gilardi-Bresson, Marine Adlanmerini, Philippe Liere, Gilles Flouriot, Rachida Guennoun, Muriel Laffargue, Jean-Michel Foidart, Françoise Lenfant, Jean-François Arnal, Raphaël Métivier, Coralie Fontaine

**Affiliations:** 1 I2MC, Institut National de la Santé et de la Recherche Médicale (INSERM) U1297, University of Toulouse 3, Toulouse, France.; 2 INSERM U1195, University Paris-Saclay, Le Kremlin-Bicêtre, France.; 3 Institut de Recherche en Santé, Environnement et Travail (Irset), INSERM UMR_S 1085, EHESP, University of Rennes, Rennes, France.; 4 Department of Obstetrics and Gynecology, University of Liège, Liège, Belgium.; 5 Institut de Génétique de Rennes (IGDR), UMR 6290, CNRS, University of Rennes, Rennes, France.

**Keywords:** Endocrinology, Vascular Biology, Cardiovascular disease, Endothelial cells, Sex hormones

## Abstract

The main estrogen, 17β-estradiol (E2), exerts several beneficial vascular actions through estrogen receptor α (ERα) in endothelial cells. However, the impact of other natural estrogens such as estriol (E3) and estetrol (E4) on arteries remains poorly described. In the present study, we report the effects of E3 and E4 on endothelial healing after carotid artery injuries in vivo. After endovascular injury, which preserves smooth muscle cells (SMCs), E2, E3, and E4 equally stimulated reendothelialization. By contrast, only E2 and E3 accelerated endothelial healing after perivascular injury that destroys both endothelial cells and SMCs, suggesting an important role of this latter cell type in E4’s action, which was confirmed using Cre/lox mice inactivating ERα in SMCs. In addition, E4 mediated its effects independently of ERα membrane-initiated signaling, in contrast with E2. Consistently, RNA sequencing analysis revealed that transcriptomic and cellular signatures in response to E4 profoundly differed from those of E2. Thus, whereas acceleration of endothelial healing by estrogens had been viewed as entirely dependent on endothelial ERα, these results highlight the very specific pharmacological profile of the natural estrogen E4, revealing the importance of dialogue between SMCs and endothelial cells in its arterial protection.

## Graphical abstract

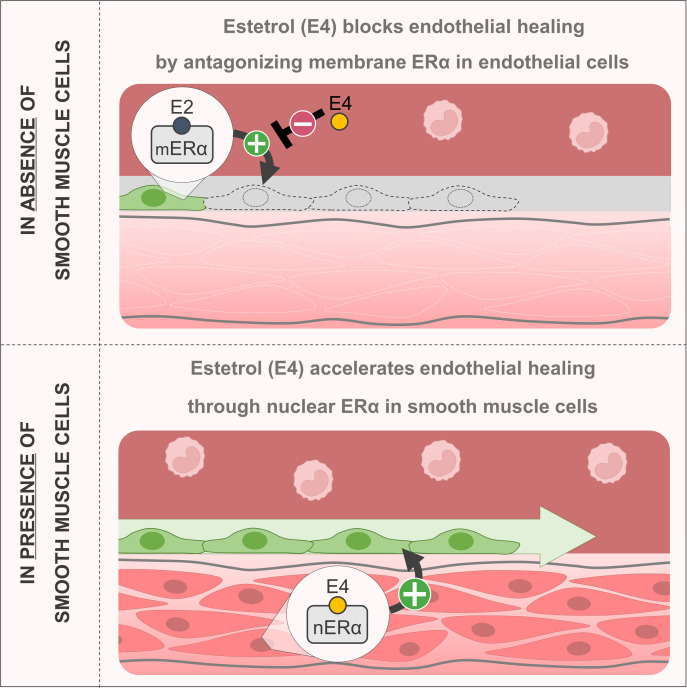

## Introduction

A large body of evidence indicates that the main endogenous estrogen, 17β-estradiol (E2), exerts beneficial effects on the endothelium (1). In particular, E2 accelerates carotid artery endothelial healing after either endovascular or perivascular injuries (2). The capacity of the endothelium to regenerate following injury is essential to ensure its role as a semipermeable barrier, and thus to prevent various vascular diseases, including atherosclerosis, restenosis, and thrombus formation (3). This beneficial effect of E2 relies on the activation of estrogen receptor α (ERα) in both endothelial and hematopoietic cells (4). As a member of the nuclear receptor superfamily, ERα is primarily considered a ligand-regulated transcription factor but besides its genomic action, a pool of ERα also localizes at the plasma membrane and mediates rapid signaling through interaction with other proteins such as endothelial nitric oxide synthase (eNOS), SRC, or several other kinases (5). Combinations of several transgenic mouse models with pharmacological tools demonstrated the pivotal role of nongenomic effects of ERα in E2-induced endothelial healing (6–9). In particular, we previously highlighted the loss of E2-mediated protection against endothelial injury in the *C451A-ERα* mouse model in which ERα is unable to localize to the plasma membrane due to a point mutation of its palmitoylation site (6). More recently, we also demonstrated the crucial role of arginine 264 of ERα, involved in PI3K and G protein interaction, in mediating this E2 effect (7).

In addition to E2, which is mainly produced by the ovaries in premenopausal women, 2 other natural estrogens, estriol (E3) and estetrol (E4) are produced during pregnancy by the placenta and the fetal liver, respectively. E3 is used to reduce genitourinary symptoms in postmenopausal women (10), and E4 was recently approved by the Food and Drug Administration and the European Medicines Agency for oral contraception and is in a phase III clinical trial for the hormone treatment of menopause (11–14). Indeed, E4 induces fewer effects on liver-derived coagulation factors than classic estrogens, and thereby could not increase the risk of venous thromboembolism (15, 16). All 3 estrogens display distinct ERα activation profiles due to differential receptor affinities, metabolism (half-life), and subfunction activation (17). Even though both E3 and E4 are commercialized for therapeutic purposes, their effects on arteries remain largely undescribed compared with E2. In 2014, we reported that E2, but not E4, was able to accelerate reendothelialization after perivascular injury of the carotid artery and that E4 was unexpectedly able to inhibit this effect (17). Along with other experiments, we concluded that despite similarities in nuclear ERα’s actions, E4 not only fails to elicit, but is even able to antagonize the membrane-initiated effects of ERα mediated by E2 (5, 17). As endothelial injury and increased endothelial turnover are key events in arterial areas prone to atheroma (18–20), lack of endothelial healing capacity could represent a disadvantage of E4 compared with E2, or even a deleterious effect in an organism with endogenous E2. Altogether, these data prompt clarification of the role of E4 in endothelial injury in a different model and exploration for the first time of the still-unknown impact of E3 in this process. To this aim, we used 2 different models of carotid artery injury to compare the involvement of each cell type (i.e., endothelial cells versus smooth muscle cells, SMCs) in response to each ligand: (a) the perivascular model induced by an electrical injury, in which both the endothelium and media are destroyed, to confirm the previous results of E2 and E4 and to evaluate the effect of E3; and (b) the endovascular model, consisting of an intraluminal injury where the endothelium is removed while SMCs are preserved. This latter endovascular model better reflects an endothelial injury induced by smoking, hyperglycemia, or hypertension, all of which lead to vascular diseases.

We found that E3, similar to E2, accelerated endothelial healing in both models. In agreement with our previous study (17), E4 did not accelerate reendothelialization in the perivascular injury model. However, surprisingly, E4 accelerated endothelial healing in the endovascular injury model. We then used a combination of transgenic mouse models harboring ERα proteins mutated for specific subfunctions or with tissue-specific deletion of ERα to assess the mechanisms underlying the particular action of E4.

## Results

### E4 accelerates endothelial healing after endovascular but not perivascular injury of the carotid artery.

**Figure 1:**
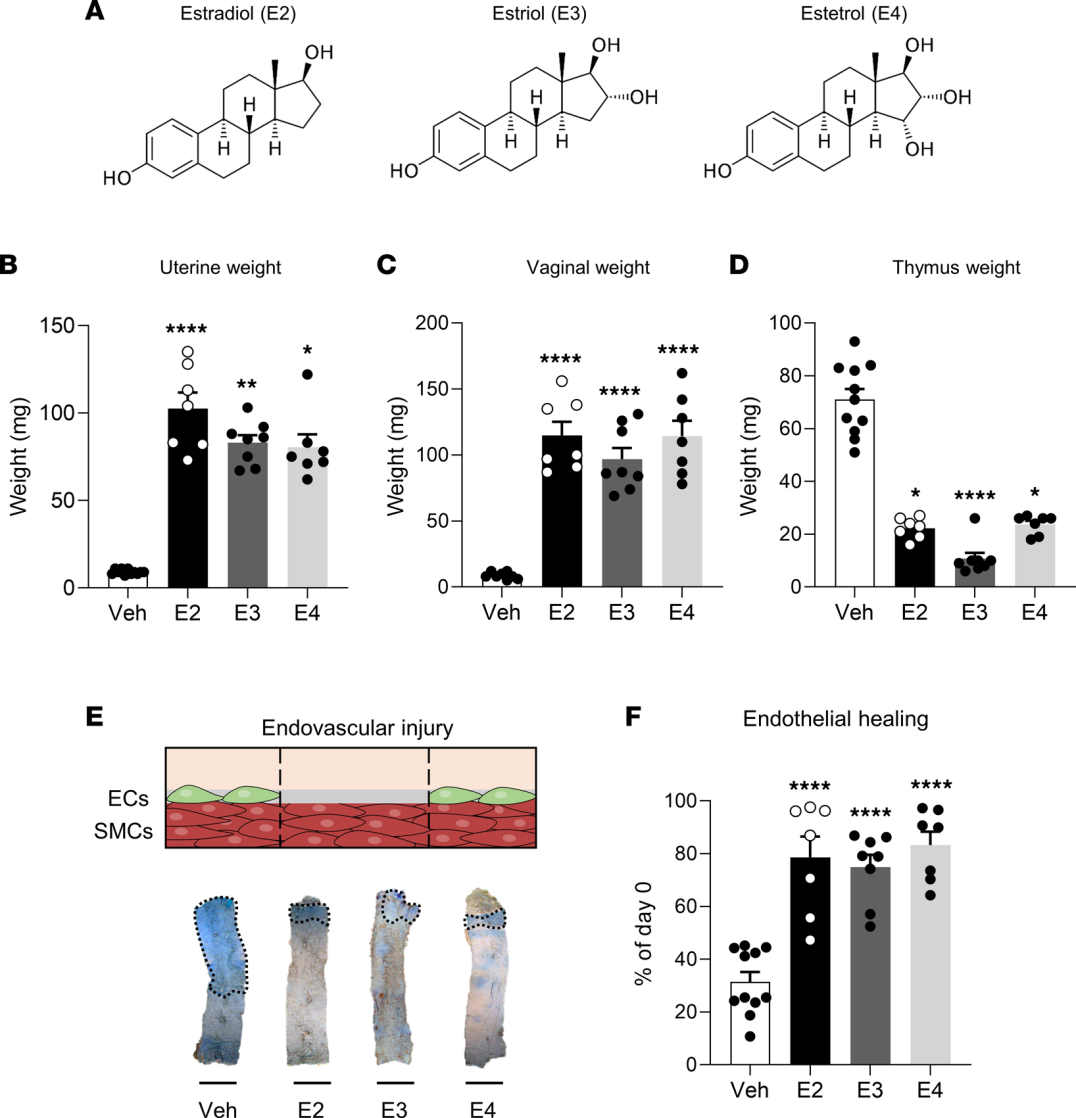
17β-Estradiol (E2), estriol (E3), and estetrol (E4) accelerate endothelial healing following carotid artery endovascular injury. Four-week-old female mice were ovariectomized and 2 weeks later implanted subcutaneously with vehicle (Veh), E2, E3, or E4 pellets for 2 weeks. Mice were then subjected to endovascular injury of the carotid artery. Carotid reendothelialization was analyzed 5 days after injury (*n* = 7–11 per group). (**A**) Chemical structures of E2, E3, and E4. (**B**) Uterine weight. (**C**) Vaginal weight. (**D**) Thymic weight. (**E**) Representative Evans blue staining of carotids with outlined deendothelialized areas (scale bar: 1 mm) and (**F**) quantitative analysis of reendothelialization, expressed as a percentage of reendothelialized area compared with day 0. ECs, endothelial cells. Results are expressed as mean ± SEM. To test the effect of the different treatments, Kruskal-Wallis test (**B** and **D**) or 1-way ANOVA (**C** and **F**) was performed. *P < 0.05, **P < 0.01, ****P < 0.0001 versus Veh-treated group.

First, to compare the effects of E2, E3, and E4 (Figure 1A) on endothelial healing, we adjusted E3 and E4 concentrations in homemade pellets to achieve estrogenic impregnation similar to that of E2, taking into account the difference in ERα affinity between estrogenic compounds (17) and using the uterotrophic effect of estrogens as an endogenous bioassay of estrogen activity. As expected, control ovariectomized mice (vehicle treated) displayed an atrophied uterus, while E2, E3, and E4 induced similar increases in uterine weight (Figure 1B). Moreover, similar vaginal impregnation (Figure 1C) and thymic atrophy (Figure 1D) were observed across treatments, supporting altogether a comparable estrogenic action of the 3 ligands under these experimental conditions. Estrogen plasma concentrations were measured by gas chromatography–tandem mass spectrometry. Importantly, no interconversion between these 3 estrogens was detected across the samples (Supplemental Table 1; supplemental material available online with this article; https://doi.org/10.1172/jci.insight.161284DS1).

**Figure 2:**
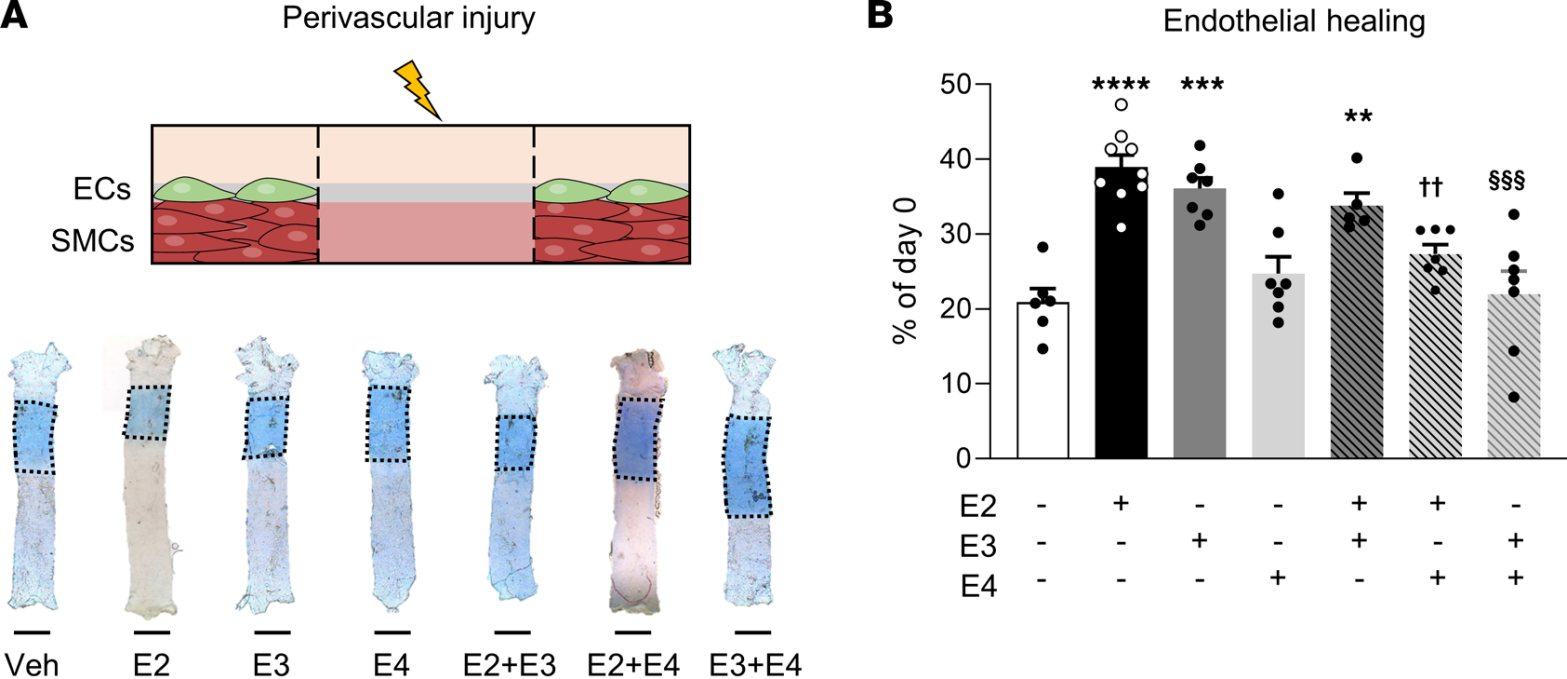
In contrast to E2 and E3, E4 does not accelerate endothelial healing after carotid artery perivascular injury. Four-week-old female mice were ovariectomized and 2 weeks later were implanted subcutaneously with vehicle (Veh), E2, E3, or E4 pellets or a combination of 2 of these estrogens for 2 weeks. Mice were subjected to perivascular injury of the carotid artery. Carotid reendothelialization was analyzed 3 days after injury (*n* = 5–9 per group). (**A**) Representative Evans blue staining of carotids with outlined deendothelialized areas (scale bar: 1 mm) and (**B**) quantitative analysis of reendothelialization, expressed as a percentage of reendothelialized area compared with day 0. ECs, endothelial cells. Results are expressed as mean ± SEM. To test the effect of the different treatments, 1-way ANOVA was performed. **P < 0.01, ***P < 0.001, ****P < 0.0001 versus Veh-treated group; ^††^*P* < 0.01 for difference between E2 and E2+E4; ^§§§^*P* < 0.001 for difference between E3 and E3+E4.

We evaluated endothelial healing by Evans blue staining after endovascular injury of the carotid artery in ovariectomized mice treated with E2, E3, or E4, a model in which SMCs are totally preserved (Figure 1E) as previously described (2). As expected, estrogenic impregnation with E2 promoted endothelial healing since quantification of reendothelialized areas showed 30% endothelial regeneration in vehicle-treated mice compared with day 0 and approximately 80% in E2-treated mice (Figure 1F). Both E3 and E4 treatments also increased endothelial healing, but no statistically significant differences in reendothelialization rates were observed between the E2-, E3-, and E4-treated groups (Figure 1, E and F). E4’s effect on endothelial healing was confirmed using VE-cadherin staining (Supplemental Figure 1). This beneficial effect of E4 contrasts at first glance with our previous work reporting the failure of E4 to promote reendothelialization after perivascular injury (17). However, in contrast to the endovascular model, the perivascular injury induces a complete decellularization of the arterial wall, including both endothelial cells and the underlying SMCs. We confirmed here that E4 is not able to accelerate endothelial healing in this perivascular model (Figure 2, A and B). In addition, we show that E3, like E2, promoted endothelial healing in this perivascular model, in striking contrast to E4. Importantly, coadministration of E4 with either E2 or E3 abrogated the accelerative effect of these 2 estrogens on endothelial regeneration (Figure 2, A and B). Altogether, these results demonstrate that the 3 endogenous estrogens, E2, E3, and E4, are able to accelerate endothelial healing in the mouse carotid artery, but the presence of underlying SMCs is specifically required for E4 to mediate this vascular action.

### ERα in SMCs is required to accelerate endothelial healing in response to E4, independently of membrane-initiated signaling.

In order to assess the mechanism underlying the particular action of E4 and to directly evaluate the role of ERα in SMCs, we used a mouse model selectively invalidated for ERα in SMCs using the inducible Cre-ER^T2^ fusion gene system under the control of the α-smooth muscle actin (α-SMA) promoter (*αSMACreER*^T2^+*ERα^lox/lox^* mice) (21). We confirmed the efficiency and specificity of ERα deletion in SMCs from the aorta and the uterus of *αSMACreER^T2^*+*ERα^lox/lox^* compared with control littermate *αSMACreER^T2^*–*ERα^lox/lox^* mice (Supplemental Figure 2). Estrogen receptor 1 (*Esr1*) gene expression was reduced by 93% in the isolated media from the aorta of *αSMACreER^T2^+ERα^lox/lox^* mice, whereas no change was observed in the adventitia (Supplemental Figure 2, A and B). Similarly, we confirmed the specific deletion of ERα in SMCs in another tissue, as ERα staining revealed specific deletion of ERα in the myometrium of the uterus from *αSMACreER^T2^+ERα^lox/lox^* mice (Supplemental Figure 2C). As we used an inducible model, we additionally confirmed that tamoxifen injections did not alter reendothelialization rates after endovascular injuries in vehicle- and E4-treated wild-type (WT) mice (Supplemental Figure 3).

**Figure 3:**
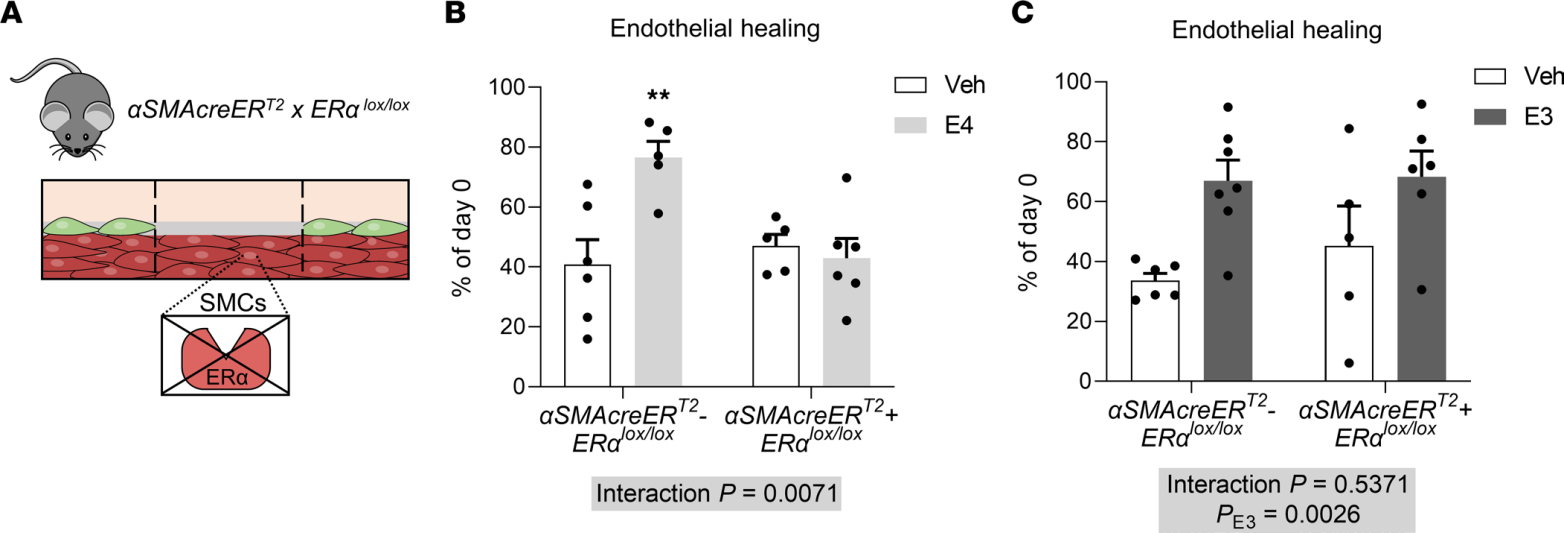
ERα in smooth muscle cells is necessary for E4’s effect on endothelial healing but dispensable for E3’s effect. (**A**) Four-week-old ovariectomized *αSMACreER*^T2^+*ERα*^lox/lox^ female mice and their respective control littermates were implanted with vehicle (Veh), E4, or E3 pellets for 2 weeks and subjected to endovascular injury of the carotid artery. Quantitative analysis of reendothelialization 5 days after injury, relative to day 0, are depicted in response to (**B**) E4 (*n* = 5–6 per group) or (**C**) E3 (*n* = 5–7 per group). Results are expressed as mean ± SEM. To test the effect of E4 and E3 treatments in each genotype, 2-way ANOVA was performed. **P < 0.01 versus Veh-treated group.

We next evaluated endothelial healing in *αSMACreER^T2^*–*ERα^lox/lox^* and *αSMACreER^T2^+ERα^lox/lox^* ovariectomized female mice supplemented or not with E4 (Figure 3A). In these mice, E4 treatment led to a similar uterine impregnation in both genotypes (Supplemental Table 2). As expected, E4 promoted reendothelialization after endovascular injury of the carotid artery in littermate control mice (40% of reendothelialization in vehicle-treated mice as compared with 80% in E4-treated mice) (Figure 3B). This accelerative effect was completely abrogated in *αSMACreER*^T2^+*ERα*^lox/lox^ mice (Figure 3B), demonstrating that ERα in SMCs is required to promote E4’s effect on endothelial healing. Altogether, unlike what we have shown for E2 (22) and E3 (Figure 3C), SMCs appear to be the main target cells for the accelerative effect of E4 on endothelial healing.

### Despite its antagonistic effect on membrane ERα signaling, E4 accelerates endothelial healing in the presence of exogenous and endogenous estrogens.

**Figure 4:**
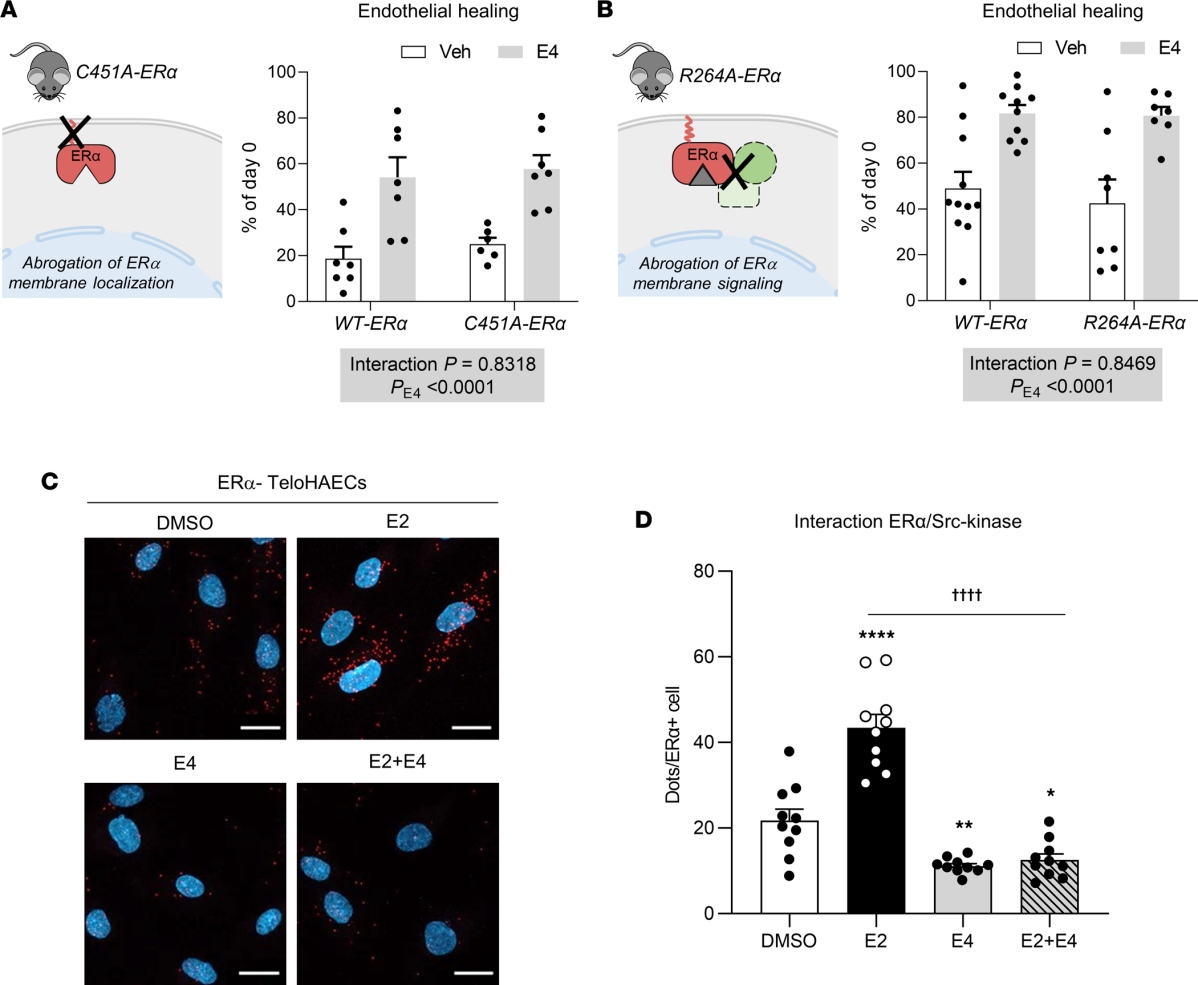
E4 does not require membrane-initiated ERα signaling to accelerate endothelial healing and antagonizes this pathway in endothelial cells. Four-week-old ovariectomized (**A**) *C451A-ERα* (*n* = 6–7 per group) and (**B**) *R264A-ERα* (*n* = 7–11 per group) female mice and their respective control WT littermates were implanted with vehicle (Veh) or E4 pellets for 2 weeks and subjected to endovascular injury of the carotid artery. Schematic representation of each mouse model and quantitative analysis of reendothelialization 5 days after injury relative to day 0 are depicted. Results are expressed as mean ± SEM. To test the effect of E4 treatments in each genotype, 2-way ANOVA was performed. (**C**) Estrogen-deprived ERα-TeloHAECs were incubated with DMSO, E2 (1 × 10^–8^ M), E4 (1 × 10^–6^ M), or a combination of E2 and E4 for 5 minutes. Proximity ligation assay for ERα-SRC interaction was performed. Interactions are represented by red dots. Nuclei were counterstained with DAPI (scale bars: 20 μm). (**D**) Quantification of the number of dots per ERα-positive cell from 1 representative experiment. The experiment was replicated 3 times. Results are expressed as mean ± SEM. To test the effect of the different treatments, 1-way ANOVA was performed. *P < 0.05, **P < 0.01, ****P < 0.0001 versus Veh-treated group. ^††††^*P* < 0.0001 for difference between E2 and E2+E4.

Since membrane ERα mediates the acceleration of reendothelialization in response to E2 (6, 22), we then decided to evaluate the role of this pathway in response to E4. To this end, we used 2 different mouse models targeting ERα membrane-initiated signaling, in which acceleration of endothelial regeneration in response to E2 was shown to be abrogated (7, 22). In *C451A-ER*α mice, ERα does not localize to the plasma membrane due to the point mutation of its palmitoylation site, leading to the loss of global membrane-initiated ER signaling. In this model, endothelial regeneration was approximately 20% in the vehicle-treated group, and E4 increased reendothelialization to 60% independently of genotype (Figure 4A), demonstrating that E4 promotes endothelial healing independently of membrane ERα. We extended this result using a second mouse model, i.e., *R264A-ER*α mice, targeting the second major amino acid involved in membrane ERα signaling (7). Similarly, we found no difference in reendothelialization rates between control and *R264A-ER*α female mice following E4 treatment (Figure 4B). In these 2 mouse models, uterine impregnation in response to E4 was similar in all genotypes (Supplemental Table 2). In addition, we used an immortalized human aortic endothelial cell line (TeloHAEC) to directly evaluate membrane ERα signaling in response to E2, E3, and E4 in endothelial cells. Since TeloHAECs (like other immortalized endothelial cell types) have no detectable ERα expression, we generated stably transduced TeloHAECs expressing full-length ERα (ERα-TeloHAECs) (Supplemental Figure 4, A–C). To evaluate membrane ERα signaling in these cells, we measured ERα interaction with the tyrosine kinase SRC using the proximity ligation assay (PLA) technique. Interaction of ERα with SRC is indicated by the presence of red dots in the cytoplasm of ERα-TeloHAECs. Importantly, no dots were detected using either only one antibody or both antibodies in TeloHAECs that do not express ERα, validating the specificity of the technique (Supplemental Figure 4, D–F). E2 and E3 increased the ERα-SRC interaction, whereas E4 failed to elicit this membrane ERα effect (Figure 4, C and D, and Supplemental Figure 5). Importantly, when administered together, E4 completely abrogated the stimulatory effect of E2 and E3 on the ERα-SRC interaction, highlighting that E4 antagonizes membrane ERα signaling in endothelial cells, as suggested in the model of perivascular injury (Figure 2).

**Figure 5:**
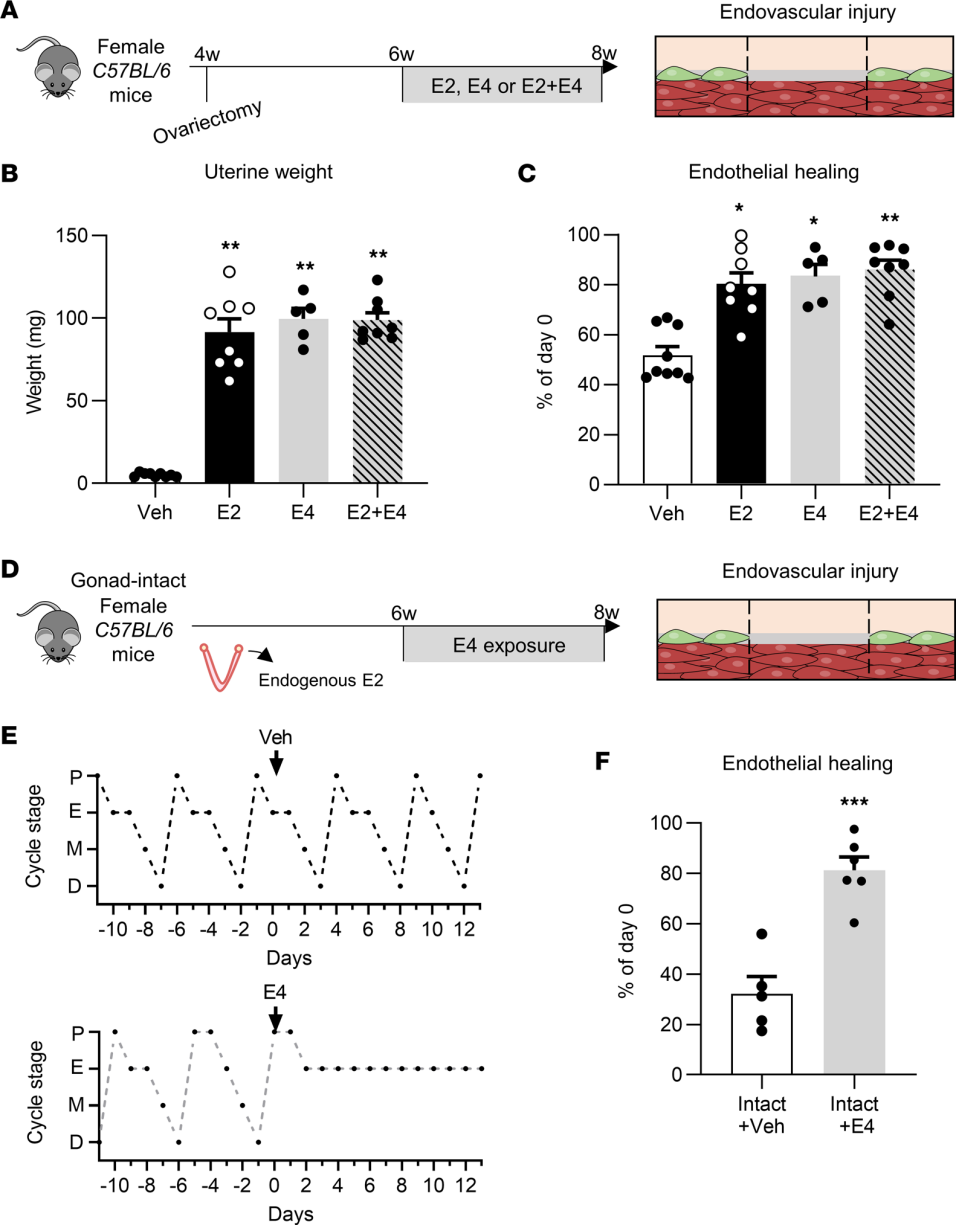
E4 still accelerates endothelial healing in the presence of exogenous and endogenous estrogens. (**A**) Four-week-old C57BL/6 female mice were ovariectomized and 2 weeks later were implanted with vehicle (Veh), E2, E4, or a combination of E2 and E4 pellets. Two weeks later, mice were subjected to endovascular injury of the carotid artery. Carotid reendothelialization was analyzed 5 days after injury (*n* = 5–9 per group). (**B**) Uterine weight. (**C**) Quantitative analysis of reendothelialization, expressed as a percentage of reendothelialized area compared with day 0. (**D**) Six-week-old gonad-intact C57BL/6 female mice were implanted with Veh or E4 pellets. Two weeks later, mice were subjected to endovascular injury of the carotid artery. Carotid reendothelialization was analyzed 5 days after injury (*n* = 5–6 per group). (**E**) Representative estrous cycles before and after Veh and E4 treatment. (**F**) Quantitative analysis of reendothelialization, expressed as a percentage of reendothelialized area compared with day 0. Results are expressed as mean ± SEM. To test the effect of the different treatments, Kruskall-Wallis test (**B** and **C**) or 2-tailed Student’s *t* test (**F**) was performed. *P < 0.05, **P < 0.01, ***P < 0.001.

The results presented above demonstrate that, on one hand, E2 and E4 act on different cell types to accelerate endothelial healing, and on the other hand E4 antagonizes E2-induced membrane ERα activity necessary for the effect of E2 on endothelial healing. As E4 is commercialized for contraception, which implies its use in the presence of endogenous estrogens, it is therefore important to assess whether its agonistic or antagonistic effects will be observed in this clinical setting. We thus decided to evaluate the impact of E4 on reendothelialization after endovascular injury of the carotid artery in the presence of exogenous or endogenous E2 (gonad-intact mice) (Figure 5). In contrast to the results obtained after perivascular injury (Figure 2), the coadministration of E4 with E2 still led to accelerated reendothelialization after endovascular injury, with no difference compared to E2 and E4 alone (Figure 5, A–C). In order to better model the use of E4 for contraception, we also administered E4 to gonad-intact female mice in which endogenous estrogens are present (Figure 5D). We validated that E4 led to the arrest of ovarian function by analyzing estrous cycles in vehicle- and E4-treated gonad-intact mice (Figure 5E). While vehicle-treated mice presented with regular estrous cycles, E4-treated mice were blocked in the estrus stage, validating our experimental model of contraception. Importantly, in gonad-intact mice, E4 accelerated endothelial healing (80% of reendothelialization compared with 30% in control mice; Figure 5F). Altogether, these results suggest that even though E4 antagonizes E2’s effects on membrane ERα in endothelial cells, the specific agonistic effect of E4 on ERα in SMCs is sufficient to mediate a beneficial endothelial healing effect compared with control mice.

### Chronic E4 and E2 treatments induce differential transcriptional programs in mouse carotid arteries.

**Figure 6:**
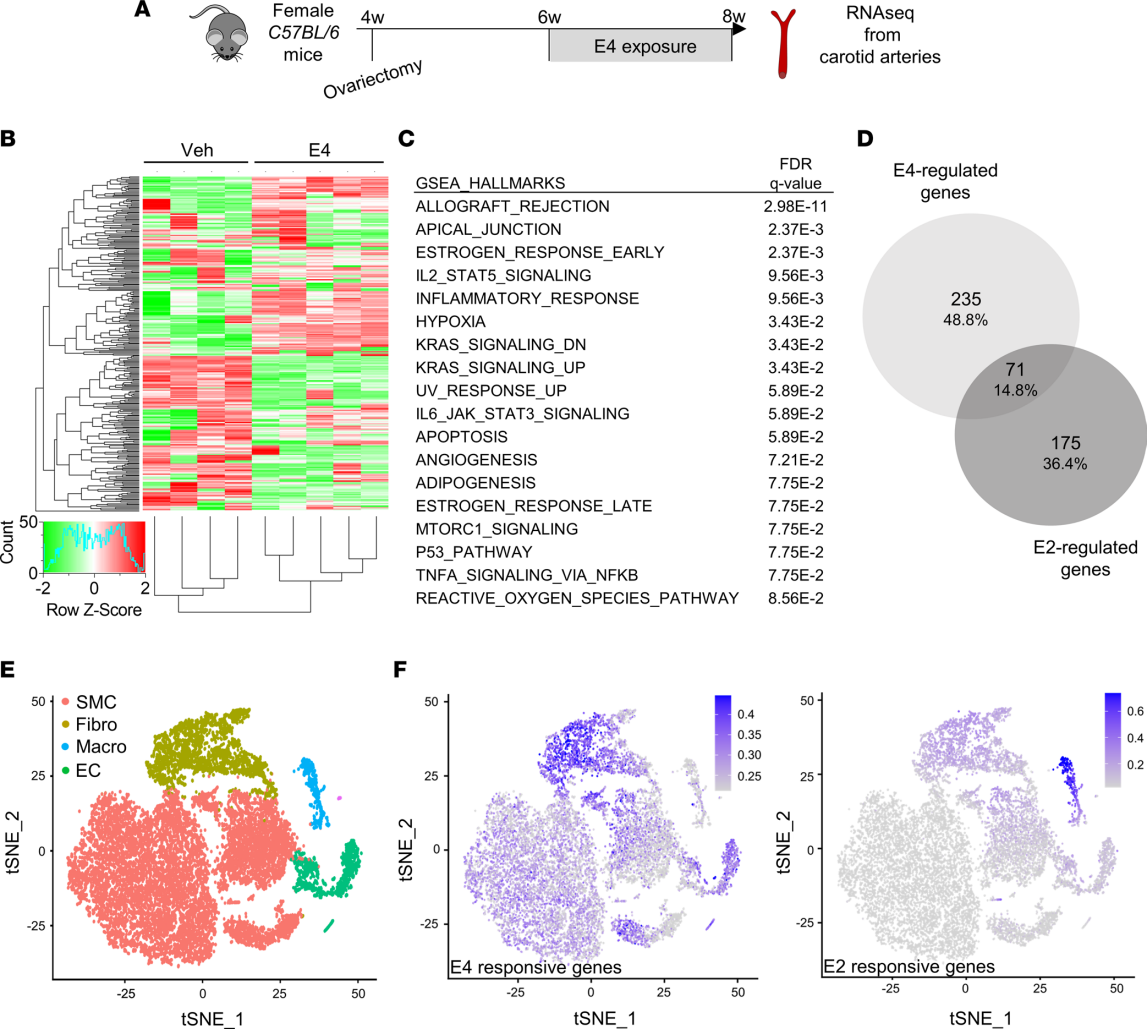
E4 displays a specific transcriptional program that differs from E2 in carotid arteries. (**A**) Four-week-old C57BL/6 female mice were ovariectomized and treated with a vehicle (Veh) or E4 for 2 weeks. RNAs were isolated from uninjured carotid arteries and sequenced (*n* = 4–5 per group). (**B**) Heatmap illustrating the relative expression values of all genes significantly regulated following E4 treatment (fold change >2 or <0.5 versus control with Benjamini-Hochberg–corrected *P* < 0.05). Hierarchical clustering regroups each sample with its corresponding treatment group. (**C**) GSEA representing the different hallmark pathways regulated by E4. Calculated false discovery rate (FDR) *q* value is given for each term. (**D**) Venn diagram representing the overlap of genes regulated by E2 and E4. (**E**) t-SNE of single-cell RNA sequencing data from carotid arteries of WT mice, organized by cell cluster (23). SMC, smooth muscle cells; Fibro, fibroblasts; Macro, macrophages; EC, endothelial cells. (**F**) Feature plots of E4-regulated genes (left) and E2-regulated genes (right) identified by RNA sequencing.

To go further in understanding the effects of E4, we next performed RNA sequencing on carotid arteries from ovariectomized female mice treated chronically with E4 (Figure 6A). This large-scale transcriptional approach was performed on noninjured carotids to avoid the complexity associated with the kinetics of endothelial healing differing across treatments. This analysis identified 306 genes as being significantly regulated by E4 in the carotid artery as compared with vehicle-treated mice (absolute log_2_[fold change] > 1, adjusted *P* value < 0.05) (Figure 6B). Functional annotation of this gene subset revealed hallmarks for early and late estrogen response (Figure 6C). Interestingly, the angiogenesis hallmark was also significantly associated with E4 treatment (Figure 6C). However, comparison of this data set with already published data on E2-regulated genes in carotid arteries in similar conditions revealed that only 14.8% of genes were commonly regulated by E2 and E4 (Figure 6D and Supplemental Figure 6). A subset of common and specific genes regulated by each ligand was validated by RT-qPCR analysis in independent experiments (Supplemental Figure 7). We then took advantage of the 2 recently published transcriptomes of single cells from a carotid artery (23, 24) to compare the transcriptional signatures of E4 and E2 with the profile of each of the cell types identified in the arterial wall (Figure 6E and Supplemental Figure 8). These results revealed that gene expression profiles in carotid arteries from mice treated with E4 and E2 substantially differ both in terms of gene regulation and of cellular targets. Indeed, in contrast to E2 response genes, which are mostly associated with monocytes/macrophages (Figure 6F and Supplemental Figure 8), genes regulated by E4 are expressed in almost all cell population subtypes that could be discriminated in carotid arteries, including SMCs (Figure 6F and Supplemental Figure 8).

**Figure 7:**
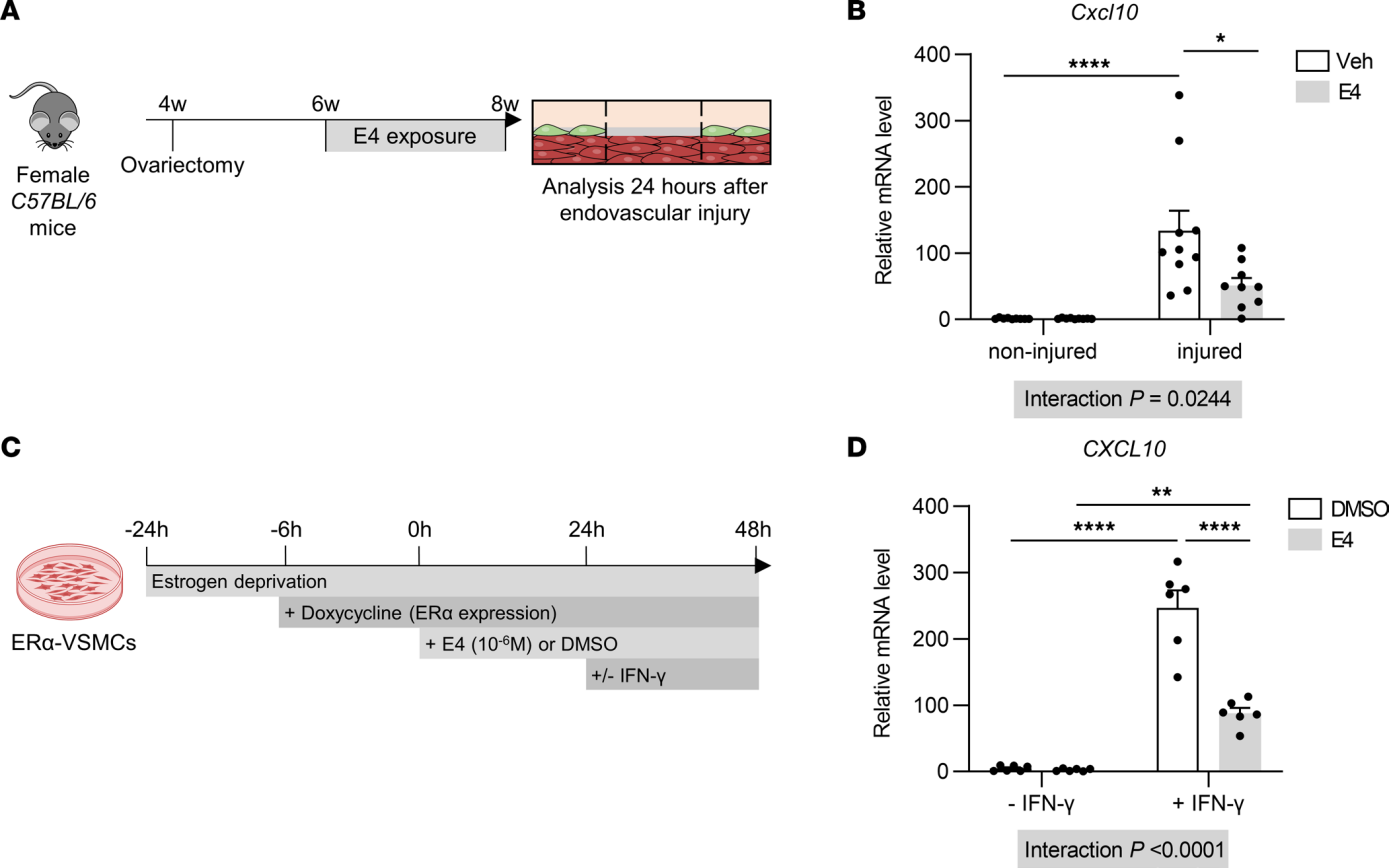
E4 treatment decreases *Cxcl10* mRNA levels in vivo in injured carotid arteries and in vitro in SMCs. (**A**) Four-week-old C57BL/6 female mice were ovariectomized and after 2 weeks of recovery were implanted with vehicle (Veh) or E4 pellets. Two weeks later, mice were subjected to endovascular injury of the carotid artery. RNAs were isolated from injured and contralateral noninjured carotid arteries 24 hours later. (**B**) RT-qPCR analysis of *Cxcl10* mRNA in noninjured and injured carotid arteries (*n* = 9–10 per group). (**C**) Stably transduced vascular SMCs expressing full-length ERα (ERα-VSMCs) were serum starved for 24 hours and then pretreated with DMSO or E4 (1 × 10^–6^ M) for 24 hours before IFN-γ stimulation. (**D**) RT-qPCR analysis of *CXCL10* mRNA in ERα-VSMCs (*n* = 6 per group from 2 independent experiments). Results are expressed as mean ± SEM. Two-way ANOVA was performed to test the effect of the different treatments. *P < 0.05, **P < 0.01, ****P < 0.0001.

Several studies highlighted that SMCs can modulate endothelial cell regeneration through paracrine effects that either accelerate or decrease reendothelialization (25). We screened for those pathways in injured carotid arteries treated (or not) with E4. mRNA levels of the chemokine pro-platelet basic protein (*Ppbp*) and genes of Notch and Hedgehog signaling were not regulated by E4 during injury (Supplemental Figure 9). However, we found that *Cxcl10* gene expression was downregulated by E4 in injured carotid arteries (Figure 7, A and B). Importantly, we recently demonstrated that this cytokine mediates a large part of the IFN-γ–dependent inhibition of reendothelialization and that its blockade improves endothelial recovery (26). To directly evaluate the effect of E4 on SMCs, we generated stably transduced vascular SMCs expressing full-length ERα (ERα-VSMCs) in which the effect of E4 on gene expression was confirmed (Supplemental Figure 10). We finally demonstrated that E4 blocks *CXCL10* induction by IFN-γ in ERα-VSMCs (Figure 7, C and D), potentially contributing to the dialogue between SMCs and endothelial cells that ultimately accounts for the acceleration of reendothelization by E4.

## Discussion

E4 and E3 are 2 natural estrogens secreted during pregnancy by the fetal liver and the placenta, respectively. E4 and E3 have weaker estrogenic activity than E2 and display bioavailability and half-life that differ from those of E2 (10). However, the physiological role of these 2 estrogens produced only during pregnancy remains unknown. The production of E4 is restricted to the fetal liver of males and females from great apes, including humans. This is particularly intriguing because so far, the liver was rather viewed as a target of estrogens in oviparous animals (27) and also in mammals (28). The conversion of the E3 into E4 by the liver suggests a peculiar position and status of this organ in the network of sex hormones. Due to these differences, it is conceivable and even likely that these 2 estrogens could have different benefit/risk profiles compared with the classic hormones (E2, ethinyl-estradiol) for their use as oral contraception or menopause treatment (10, 15, 29). In particular, E4 is marketed for oral contraception and is currently being evaluated in phase III clinical studies as a new hormone therapy for menopause, as it was found to have limited effects on coagulation factors in the liver of women, thus leading one to expect fewer thrombotic events (15, 16). E4 significantly improves vasomotor and genitourinary menopausal symptoms and prevents bone demineralization (15). Herein, we demonstrated for the first time to our knowledge that E2, E3, and E4 equally accelerate endothelial healing after endovascular injury of the carotid artery in which medial SMCs are preserved. In contrast, only E2 and E3, but not E4, induced endothelial healing after perivascular injury.

This work emphasizes the importance of using several models while studying vascular injuries. We extensively used the perivascular model of carotid artery injury, as precise deendothelialization can be performed rather easily despite the small size of this vessel in mice (20). This model led us to characterize the accelerative effect of E2 as a phenomenon purely dependent on ERα expression in endothelial and hematopoietic cells (4, 30), and we then demonstrated that membrane ERα signaling is necessary (6, 7). Using Katzenellenbogen’s elegant tools, it appeared that activation of membrane ERα was even sufficient to accelerate endothelial healing in the same perivascular model (8, 9). More recently, we confirmed that both endothelial/hematopoietic ERα and membrane ERα subfunctions are necessary to induce endothelial healing in response to E2 using the endovascular model of vascular injury that preserves SMCs (22). In this study, we demonstrate that membrane ERα is totally dispensable for E4’s effects on endothelial healing since the acceleration of endothelial healing after endovascular injury is completely preserved in the 2 genetically modified mouse models targeting membrane ERα localization (*C451A-ERα*) and signaling (*R264A-ERα*).

In addition, using the *αSMAcreER^T2^*+*ERα^lox/lox^* mouse model, we demonstrated that ERα in SMCs is necessary to mediate this E4 vascular effect in striking contrast to E2’s effect, which acts through endothelial/hematopoietic ERα to induce similar effects (4, 22). Large-scale transcriptomic analysis also revealed substantial differences in gene regulation and associated cell signature between carotid arteries from mice treated with E4 or E2. Hence, in addition to the poor overlap existing between the E2- and E4-regulated genes, we found that these genes were mostly expressed in different cell types in single-cell RNA sequencing data sets originating from ligated or normal carotid arteries. More precisely, E2-responsive genes are expressed at significantly higher levels in monocytes, macrophages, and fibroblasts than in the other discriminated cell types. In contrast, E4-regulated genes were found to be expressed in almost all cell types from both single-cell studies, except macrophages and T cells. Altogether, these results suggest that E4 could act as a natural selective estrogen receptor modulator, targeting different cell types than E2 to achieve a similar vasculoprotective effect.

This study highlighted the contribution of endothelial cell–SMC dialogue to the E4-mediated endothelial regenerative effects after carotid injury. There is an increasing body of literature that reports the role of paracrine effects exerted by SMCs on reendothelialization and several factors are implicated in endothelial cell–SMC communications, including chemokines, cytokines, and microRNAs (25). Inhibition of *Cxcl10* by E4 observed in injured carotid arteries as well as in IFN-γ–activated SMCs could contribute to the mechanism underlying E4’s accelerative effect on endothelial healing. Indeed, CXCL10 inhibits proliferation and migration of endothelial cells in vitro (31–33) and its blockade in vivo improves endothelial recovery after carotid artery injury (26).

The effects of E4 on cardiovascular risk are of utmost importance because the benefit/risk ratio of treatment for climacteric symptoms at menopause is problematic, as demonstrated by the Women’s Health Initiative (WHI) study (34). Indeed, it is now clear that, 20 years after the WHI study, the spectrum of the cardiovascular effects of estrogens is beneficial when given early and/or between 50 and 60 years old (34). We previously demonstrated that E4 was able to prevent atheroma (13), angiotensin II–induced hypertension (35), postinjury neointima formation (21), and favor blood flow remodeling (35). Thus, the present work adds a new facet of arterial protection against endothelial injury when the underlying SMCs are present, which is the case in physiology and pathophysiology. In the context of menopause modeling, one limitation of the present study is that these experiments were performed in young mice and the WHI study (34) revealed that timing and age can be determinants for the arterial protection conferred by estrogens. These aspects should be kept in mind in future studies.

However, the main goal of the present work was to investigate the arterial effects of E4 in a situation involving contraception, i.e., in young mice. E4 was characterized as an antagonist of membrane ERα, as it was able to block the accelerative effect of E2 in this perivascular model (17). Accordingly, in vitro, we were able to directly demonstrate that E4 antagonizes the membrane ERα-SRC interaction in endothelial cells. We confirm here the efficient blocking of the accelerative effect of E2 by E4, and extend this paradigm to E3 that was also inhibited by E4. Thus, we explored the possible interaction of E4 with endogenous estrogens, mainly E2, in non-ovariectomized (intact) mice using endovascular injury, which better reflects endothelial injury leading to vascular diseases. We found that E2, E4, and their combination were similarly efficient in endothelial healing and that E4 accelerated reendothelialization also in the presence of endogenous E2. As E4 is now available for women’s oral contraception, it is quite reassuring that, thanks to these experiments, E4 does not impair endothelial healing; on the contrary, it promotes endothelial healing in a mouse model mimicking a contraceptive treatment. Therefore, together with its lesser impact on coagulation factors (15, 16) strongly suggesting safety in terms of venous thromboembolic risk, E4 could provide arterial protection and thus appears a promising option for contraception or hormonal treatment of menopause.

## Methods

### Mice.

WT female mice with a C57BL/6J background were purchased from Charles River Laboratories. *αSMACreER^T2^ERα^lox/lox^*, *C451A-ERα*, and *R264A-ERα* mouse lines were generated on the C57BL/6 background at the Mouse Clinical Institute (MCI, Strasbourg, France), as previously described (6, 7, 21). Genetically modified female mice were systematically compared to their WT littermates. Throughout all protocols, mice were housed at the animal facility of the Faculty of Medicine of the University of Toulouse, in a temperature-controlled room with a 12-hour light/dark cycle and maintained with access to food and water ad libitum.

Bilateral ovariectomy was performed at 4 weeks of age after intraperitoneal injection of tiletamine/zolazepam (Zoletil, 100 mg/kg, Virbac) and xylazine (Rompun, 10 mg/kg, Bayer HealthCare Animal Health). Prior to treatment, *αSMACreER*^T2^–*ERα^lox/lox^* (control mice) and *αSMACreER*^T2^+*ERα^lox/lox^* mice were injected over 5 days with tamoxifen (1 mg/mouse/day; Sigma-Aldrich) starting at 5 weeks of age to induce activation of Cre recombinase.

In all experiments, prior to sacrifice, mice were anesthetized with an intraperitoneal injection of tiletamine/zolazepam (Zoletil, 200 mg/kg) and xylazine (Rompun, 20 mg/kg) and euthanized by bilateral thoracotomy.

### Pellet preparation and estrogenic treatments.

To deliver estrogens chronically, E2 (Sigma-Aldrich), E3 (Sigma-Aldrich), and E4 (Mithra) were thoroughly mixed with cholesterol (Sigma-Aldrich) in powder form and compacted to obtain pellets, as previously described (13, 36). At the age of 6 weeks, ovariectomized or gonad-intact mice were implanted subcutaneously with pellets releasing E2 (75 μg/pellet), E3 (750 μg/pellet), E4 (1 mg/pellet), or vehicle (cholesterol only) for 2 weeks. The surgical procedure was performed under anesthesia by inhalation of 3% isoflurane and maintained with 1.5%–2% isoflurane mixed with 100% O_2_.

### Assessment of estrous cycle.

In gonad-intact female mice, vaginal smears were performed with PBS daily to analyze estrous cyclicity, starting 10 days before vehicle or E4 pellet implantation and for 14 days after. Vaginal cytology was assessed under a Leica DM2500 microscope and LAS X software.

### Mouse carotid injuries and quantification of reendothelialization.

Endovascular injury of the carotid artery was performed on mice as described previously (2), after 2 weeks of estrogenic treatment. Briefly, animals were anesthetized by an intraperitoneal injection of tiletamine/zolazepam (Zoletil, 100 mg/kg) and xylazine (Rompun, 10 mg/kg). The right common carotid artery was exposed and blood flow was locally restricted. The external carotid was ligated distally and incised. A 0.35 mm diameter flexible wire with a 0.25 mm tip was advanced and pulled back 3 times into the common carotid artery (on 5 mm length). The external carotid was then ligated proximally, and blood flow was restored in internal and common carotids.

Perivascular injury of the carotid artery was performed on mice as described previously (2), after 2 weeks of estrogenic treatment. Briefly, animals were anesthetized by an intraperitoneal injection of tiletamine/zolazepam (Zoletil, 100 mg/kg) and xylazine (Rompun, 10 mg/kg). The right common carotid artery was isolated and electrical injury was applied (on 3 mm length) with a bipolar microregulator.

Five days (endovascular) or 3 days (perivascular) after injury, endothelial regeneration was evaluated by staining the denuded areas with Evans blue dye. Five minutes before euthanasia, mice were injected retro-orbitally with 50 μL of 4% Evans blue dye (sc-203736, Santa Cruz Biotechnology) diluted in PBS. Mice were perfused with PBS and the right common carotid artery was dissected from the aortic arch to the carotid bifurcation and fixed with 10% phosphate-buffered formalin (Electron Microscopy Sciences) for 20 minutes. Arteries were opened longitudinally and mounted with Kaiser’s glycerol gelatin (Merck). Images were acquired and quantified using a Leica DM2500 microscope and LAS X Software. The percentage of reendothelialization was calculated relative to the initial deendothelialized area (day 0).

### Lentivector construction and production.

The cDNA encoding human ERα was subcloned into pENTR1A (Thermo Fisher Scientific) by the transfer of a BamHI/EcoRI fragment from pCR3.1-hERα66. The transfer into the destination vector pInducer20 was achieved using the Gateway LR-clonase enzyme mix. Lentiviruses were produced by the vectorology plateau at INSERM UMR1037 (Toulouse). Briefly, endotoxin-free midiprepped DNA was used to produce lentivirus particles in HEK293FT cells after a CaCl_2_/HEPES-mediated triple transfection together with the plasmids p8.71 and pVSVg, using respectively a ratio of 2:2:1 of the 3 plasmids. Viral production titers were determined by ELISA (Innotest HIV p24, Fujirebio). Functional viral titers were assessed in HT1080 cells with serial dilutions and scored for GFP expression by flow cytometric analysis on a MACSQuant 10 analyzer (Miltenyi Biotec).

### Cell culture, transduction, and treatments.

TeloHAECs (hTERT-immortalized HAECs, gift from A. Negre-Salvayre, INSERM U1297, University of Toulouse) were cultured in Endothelial Cell Growth Medium (PromoCell) at 37°C in 5% CO_2_. VSMCs (human, SV40T immortalized, isolated from mesenteric arteries; gift from A. Negre-Salvayre) were cultured in DMEM containing Glutamax (Sigma-Aldrich) supplemented with 10% heat-inactivated FBS (Gibco), penicillin (100 U/mL), and streptomycin (100 μg/mL) at 37°C in 5% CO_2_. TeloHAECs and VSMCs, which do not express ERα, were transduced with the inducible ERα expression lentivector (ERα-TeloHAECs and ERα-VSMCs) in transduction medium (OptiMEM-GlutaMAX, Life Technologies) containing 5 μg/mL protamine sulfate for 6 hours. ERα-TeloHAECs and ERα-VSMCs were then selected and maintained in media containing 0.3 mg/ml neomycin (Sigma-Aldrich). For PLAs, TeloHAECs and ERα-TeloHAECs were plated on 14 mm diameter coverslips in 6-well plates (250,000 cells/well) and grown in phenol red–free, serum-free Endothelial Cell Basal Medium (PromoCell) for 24 hours before the experiment. To induce ERα expression, cells were treated for 6 hours with 0.5 μg/mL doxycycline and then treated with DMSO vehicle, 10 nM E2, 1 μM E3, and/or 1 μM E4 for 5 minutes. For gene expression analysis, ERα-VSMCs were plated in 6-well plates. After reaching 90% confluence, VSMCs were grown in phenol red–free DMEM (Sigma-Aldrich) supplemented with 2.5% charcoal-stripped FBS (Gibco) for 24 hours prior to E4 treatment. Six hours before E4 treatment, cells were treated with 0.1 μg/mL doxycycline to induce ERα expression. Then, VSMCs were pretreated with DMSO vehicle or 1 μM E4 for 24 hours before adding recombinant IFN-γ (10 ng/mL, Peprotech). After an additional 24 hours, cells were washed twice with cold PBS and 500 μL TRIzol (Ambion) reagent was added to the wells.

### Immunofluorescence.

To determine ERα expression, cells were fixed with 4% paraformaldehyde for 10 minutes, permeabilized in PBS containing 0.1% Triton X-100 for 10 minutes, and incubated in PBS containing 1% BSA (Sigma-Aldrich), 2% FBS (Gibco), and 10% normal donkey serum (Jackson ImmunoResearch) for 1 hour. Coverslips were incubated with anti-ERα rabbit monoclonal (1:250; Abcam, ab16660) primary antibody overnight at 4°C. After washing, coverslips were incubated with Alexa Fluor 488–conjugated anti-rabbit secondary antibody (1:1000; Jackson ImmunoResearch, 711-545-152) for 45 minutes at room temperature. Nuclei were stained with DAPI (0.5 μg/mL) and coverslips were mounted with Dako Mounting Medium (Agilent Technologies). Microscopy imaging was performed with a Leica DMi8 microscope at ×40 magnification.

### Protein preparation and detection by Simple Western capillary analysis.

Total cell lysates were prepared in RIPA buffer (Sigma-Aldrich) supplemented with protease inhibitors (Roche). Simple Western analyses were performed according to the Protein Simple user manual. In brief, cell lysate samples (final concentration, 0.5 mg/mL) were mixed with a fluorescent master mix (Protein Simple) and heated at 95°C for 5 minutes. The primary antibody, validated for Simple Western, was diluted in antibody diluent (Protein Simple). The molecular weight markers, samples, protein normalization reagent, blocking reagent, primary antibody (anti-ERα rabbit monoclonal primary antibody [1:50; Cell Signaling Technology, 13258S]), HRP-conjugated secondary antibody, and chemiluminescent substrate (luminol/peroxide) were dispensed into designated wells in a manufacturer-provided microplate. The plate was loaded into the instrument (Jess, Protein Simple) and proteins were drawn into individual capillaries on a 25-capillary cassette (12–230 kDa) (Protein Simple). Data were analyzed with Compass software (Protein Simple). Normalization against total proteins in the capillary was achieved using the included Protein Normalization (PN) assay reagent.

### PLA.

The PLA (Duolink, Sigma-Aldrich) was used to detect ERα’s interaction with SRC, according to the manufacturer’s instructions. Briefly, after treatment, cells were fixed with 4% paraformaldehyde for 10 minutes and then permeabilized in PBS containing 0.2% Triton X-100 for 10 minutes. Coverslips were incubated with anti-ERα rabbit polyclonal (1:500; Santa Cruz Biotechnology, sc-543) and anti-SRC mouse monoclonal (1:500; Santa Cruz Biotechnology, sc-8056) primary antibodies overnight at 4°C. After washing, cells were incubated with anti-rabbit PLUS and anti-mouse MINUS probes coupled to oligonucleotides (Sigma-Aldrich) for 1 hour at 37°C in a humidity chamber, followed by a ligation step for 30 minutes and a rolling-circle amplification (RCA) reaction in which fluorescently labeled oligonucleotides were hybridized to the RCA product. Coverslips were then stained for total ERα as described above in the *Immunofluorescence* section. The cover slides were mounted in Duolink II mounting medium with DAPI (Sigma-Aldrich). Images were obtained with a Zeiss LSM900 confocal microscope at ×63/oil magnification and processed using Zen software. PLA dots were analyzed using ImageJ software (NIH). The plugin “Counter cells” allows analyzing dots and cell numbers. In each condition, red dots were scored in ERα-positive cells from 10 microscopic fields.

### RNA sequencing and analysis.

Total RNA was isolated from uninjured carotid arteries through the phenol-chloroform method using TRIzol (Ambion) reagent. Quality of RNA samples was determined using a Fragment Analyzer Instrument. All selected samples had RNA quality numbers above 7.2. mRNA sequencing libraries were prepared according to Illumina’s protocols using the Illumina TruSeq Stranded mRNA kit (reference no. 20020595), as previously described (22). Sequencing was paired-end (2 × 150 bp) and performed on an Illumina NovaSeq sequencer at the Integragen company platform. The reads were first trimmed for adapters and low-quality ends by the Trim Galore! algorithm (-- t, -q, -e, --length 20) available on the Galaxy web server (https://usegalaxy.org/). Subsequent informatics processes of the sequenced reads were locally done under python and R environments, as previously described (22). Genes were designated as differentially regulated when their fold change was greater than 2 or less than 0.5 with a Benjamini-Hochberg–adjusted *P* value of less than 0.05. Functional annotations were made using the Gene Set Enrichment Analysis (GSEA) program (v4.0.3) (37) interrogating the Molecular Signatures Database (MSigDB) hallmarks (38). Vehicle, E2, and E4 data sets were produced in the same experiment. All data and materials (Fastq files) have been made publicly available at the NCBI Gene Expression Omnibus (GEO) portal (39) and can be accessed with accession number GSE154268.

### Analysis of single-cell RNA sequencing experiments.

Fastq files containing reads for carotid experiments (23) were downloaded from the NCBI SRA database (https://www.ncbi.nlm.nih.gov/sra) (access numbers: SRR13932927, SRR13932928, SRR13932929, SRR13932930, SRR14242380, and SRR14242381). Alignment onto the mm10 mouse genome, UMI counting, and quality control steps were done under the CellRanger (v6.1.2) --count pipeline from 10× Genomics. The generated h5 files were then processed with Seurat R package (v3) (40). We removed low-quality cells by selecting those containing over 1.5-fold more and 1.5-fold less than the minimum or maximum of the detected RNA features, respectively. We also excluded those with lower than 10% of their features as being mitochondrial genes. Data sets were normalized and scaled to the data corresponding to the 5,000 most variable features. Principal component analysis (PCA) was performed on the scaled data, and the dimensionality of the data set was estimated by the Elbow heuristic method. Clustering of the cells in different communities was done by applying the Louvain algorithm implemented in Seurat R, and was visualized by t-distributed stochastic neighbor embedding (t-SNE). To visualize the cells expressing the different sets of genes regulated by E2 or E4 identified in our RNA sequencing, we first identified those common to the marker genes of the different cell communities, obtained by applying the MAST R package (41). We then projected these filtered lists on the t-SNE map by using the FeaturePlot feature of Seurat R with thresholds min.cutoff = “q10” and max.cutoff = “q90.”

### Statistics.

Results are expressed as mean ± SEM. Statistical analyses were performed using Prism 9 (GraphPad Software). A *P* value of less than 0.05 was considered significant. Gaussian (normal) distribution was determined using the Shapiro-Wilks normality test. For normally distributed populations, 2-tailed Student’s *t* test (2 groups) or 1-way ANOVA followed by Bonferroni’s post test (3 or more groups) was conducted. For data that failed normality testing, the Mann-Whitney test (2 groups) or Kruskal-Wallis with Dunn’s post test (3 or more groups) was performed. To test the respective roles of treatment and genotype, 2-way ANOVA was performed. Exact number (*n*), precise *P* values, and statistical tests used in each experiment are described in the supplemental material. When representative images are shown, the selected images were those that most accurately represented the average data obtained in all the samples.

### Study approval.

All procedures involving animals were performed in accordance with the principles and guidelines established by the National Institute of Health and Medical Research (INSERM) and were approved by the local Animal Care and Use Committee (CEEA122, Toulouse, France) and the French Ministry of Higher Education, Research and Innovation (Paris, France). The investigation conforms to the directive 2010/63/EU of the European parliament.

## Author contributions

C. Fontaine, JFA, RM, JMF, ML, and FL conceived and designed this study. MD, RZ, MB, NFS, C. Febrissy, HL, MA, PL, GF, and RG conducted experiments and acquired and analyzed data. SGB assisted with experiments. MD, JFA, RZ, MB, RM, and C. Fontaine drafted the manuscript and assembled figures. All authors discussed the results and commented on the manuscript.

## Supplementary Material

Supplemental data

## Version 1 02/02/2023

In-Press Preview

## Version 2 03/08/2023

Electronic publication
