# Supplementary material for: The different natural estrogens promote endothelial healing through distinct cell targets
Source: JCI Insight. 2023 Feb 2;8(5):e161284. doi: 10.1172/jci.insight.161284 (PMC10070101; doi:10.1172/jci.insight.161284)
Supplement: Supplemental data [file jciinsight-8-161284-s029.pdf]

SUPPLEMENTAL DATA

The different natural estrogens promote endothelial healing through distinct cell targets

Morgane Davezac[1], Rana Zahreddine[1], Melissa Buscato[1], Natalia F. Smirnova[1], Chanaelle Febrissy[1], Henrik Laurell[1], Silveric Gilardi-Bresson[1], Marine Adlanmerini[1], Philippe Liere[2], Gilles Flouriot[3], Rachida Guennoun[2], Muriel Laffargue[1], Jean-Michel Foidart[4], Françoise Lenfant[1], Jean-François Arnal[1], Raphaël Métivier[5] and Coralie Fontaine[1].

1 I2MC, Institut National de la Santé et de la Recherche Médicale (INSERM) U1297, University of Toulouse 3, Toulouse, France

2 INSERM U1195, University Paris-Saclay, Le Kremlin-Bicêtre, France

3 Univ Rennes, EHESP, Irset (Institut de Recherche en Santé, Environnement et Travail) – INSERM, UMR_S 1085, Rennes, France

4 Department of Obstetrics and Gynecology, University of Liège, Liège, Belgium

5 CNRS, Univ Rennes, IGDR (Institut de Génétique De Rennes) – UMR 6290, F-35000 Rennes, France

[#] Corresponding author

Jean-François Arnal

INSERM/UPS U1297 - I2MC
Institut des Maladies Métaboliques et Cardiovasculaires
1 avenue Jean Poulhès, BP 84225
31432 Toulouse Cedex 4
Tel: +33 5 31 22 40 98
E-mail: jean-francois.arnal@inserm.fr

SUPPLEMENTAL FIGURES

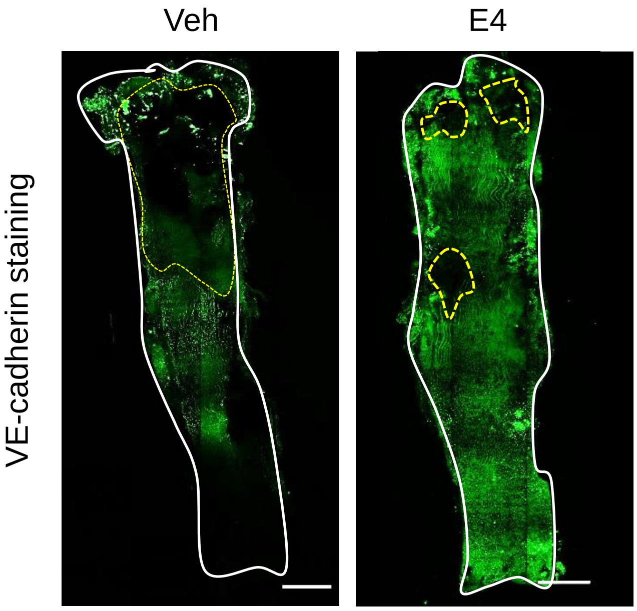

Supplemental Figure 1: Representative VE-cadherin staining of *en face* carotid artery after endovascular injury. The carotid artery is outlined in white. VE-cadherin staining is represented in green. Non-stained deendothelialized areas are outlined in yellow (scale bar, 500 µm).

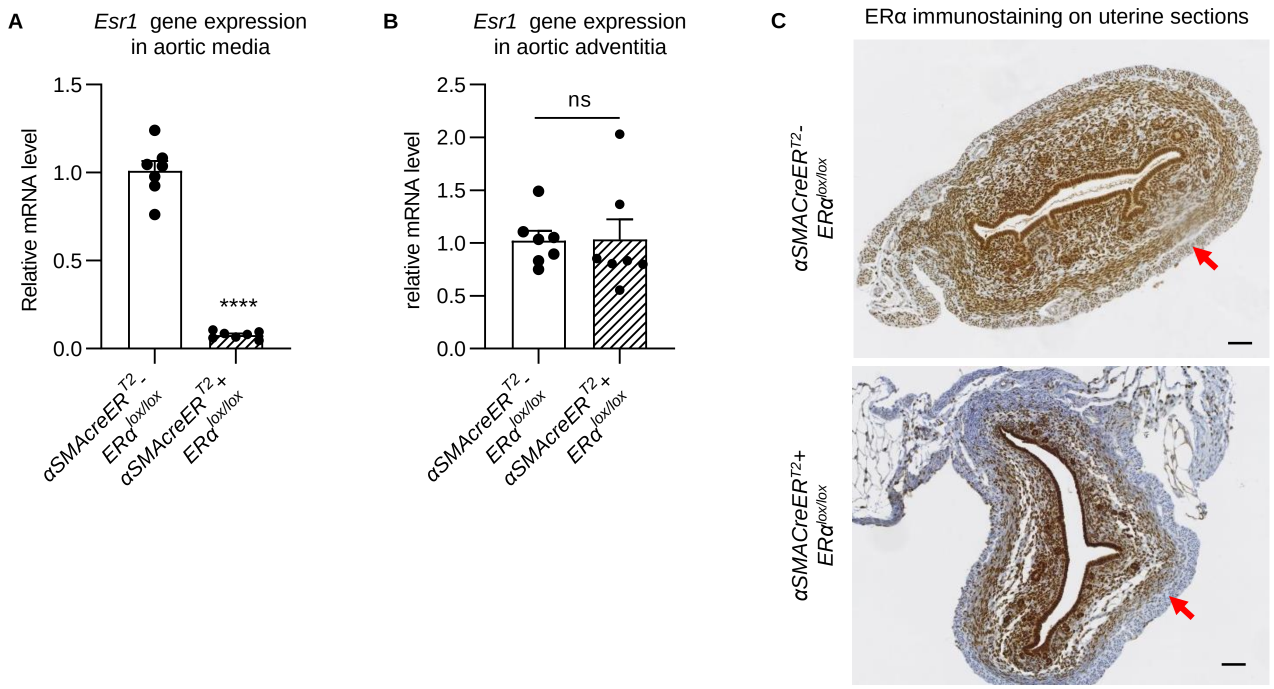

Supplemental Figure 2: Efficiency and specificity of ERα deletion in smooth muscle cells. Female *αSMACreER*^{T2}+*ERα*$^{lox/lox}$ mice and their control littermates (*αSMACreER*^{T2}-*ERα*$^{lox/lox}$) were ovariectomized and injected with tamoxifen to induce cre recombinase. ERα (*Esr1*) mRNA levels were analyzed in media (**A**) and aventitia (**B**) isolated from the aortas (n = 7 per group). Representative ERα staining (brown) on transverse uterine sections is shown in (**C**), the arrow indicates the myometrum (scale bar: 100 µm; n = 6 uteri per group were analyzed). Results are expressed as mean ± SEM. To test difference between genotypes a Student *t*-test (**A**) or a Mann-Whitney test (**B**) was performed (****P<0.0001, ns: non significant).

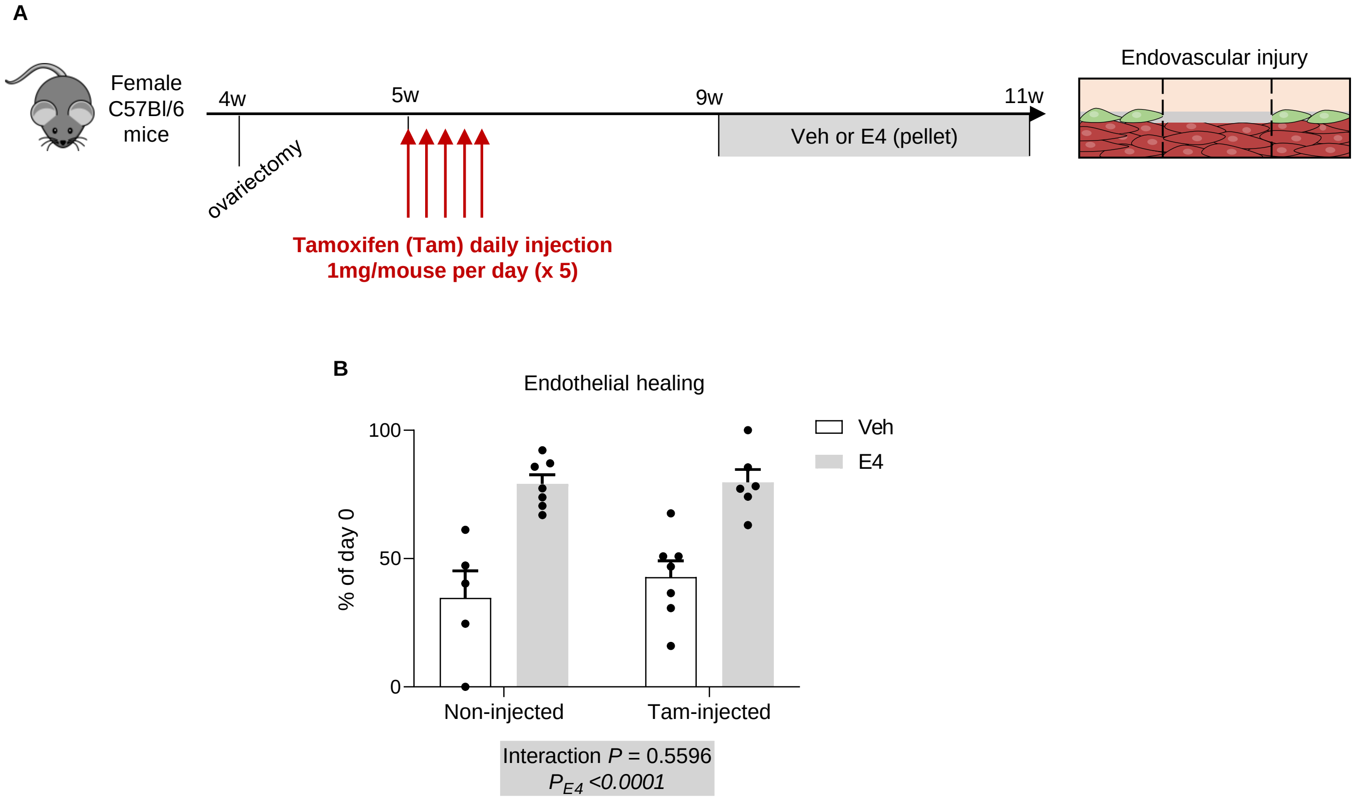

Supplemental Figure 3: Tamoxifen injections (1 mg/mouse per day during 5 days) do not impact the effect of E4 on endothelial healing. (**A**) Bilateral ovariectomy was performed at 4 weeks of age. One week after, mice were injected (or not) during 5 days with tamoxifen (1 mg/mouse per day). After a washout period of three weeks (at week 9), mice were implanted subcutaneously with pellets releasing E4 (1mg/pellet) or a vehicle (cholesterol only) for 2 weeks. Mice were then submitted to endovascular injury of the carotid artery. Carotid reendothelialization was analyzed 5 days post-injury. (**B**) Quantitative analysis of reendothelialization relative to day 0 is depicted (*n* = 5–7 per group). Results are expressed as means ± SEM. To test the effect of the different treatments (Tam injection and E4 treatment), a 2-way ANOVA test was performed.

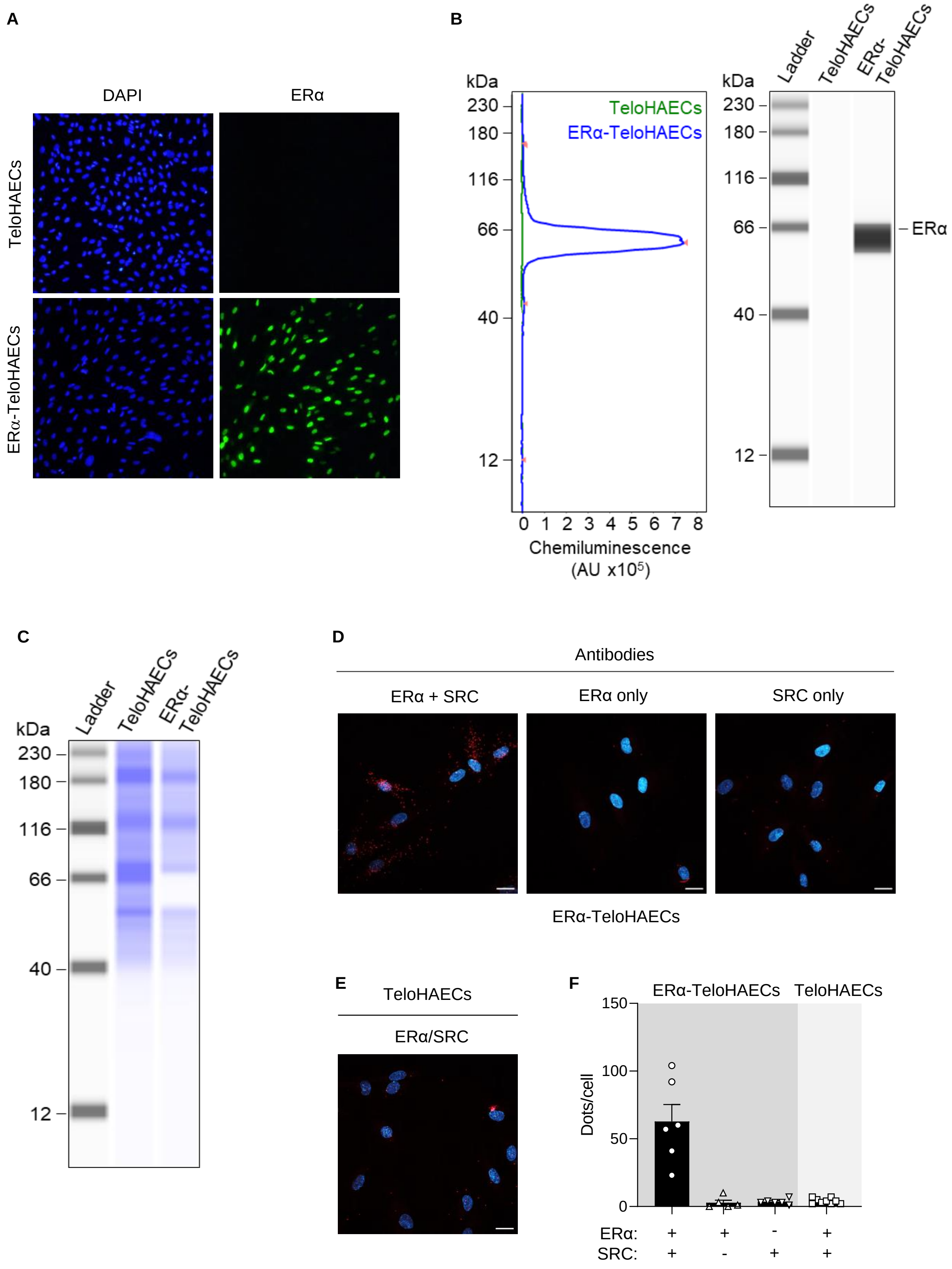

Supplemental Figure 4: Validation of Proximity Ligation Assay (PLA) technique in endothelial cells with stable ERα expression. (**A**) Representative ERα (green) and DAPI (blue) staining on control endothelial cells (TeloHAECs) or endothelial cells expressing ERα (ERα-TeloHAECs); Obj: X40. (**B**) Representative ERα protein expression and (**C**) total proteins analysed by Simple Western in TeloHAECs or ERα-TeloHAECs. (**D**) Representative images of PLA performed with ERα and SRC antibodies or with single antiblodies in ERα-TeloHAECs incubated with E2 for 5min. (**E**) Representative image of PLA performed with ERα and SRC antibodies in TeloHAECs incubated with E2 for 5min. The detected dimers are represented by red dots. Nuclei were counterstained with DAPI (blue) (Scale bar: 20µm). (**F**) Quantification of the number of dots per cell. The experiments were reproduced 2 times.

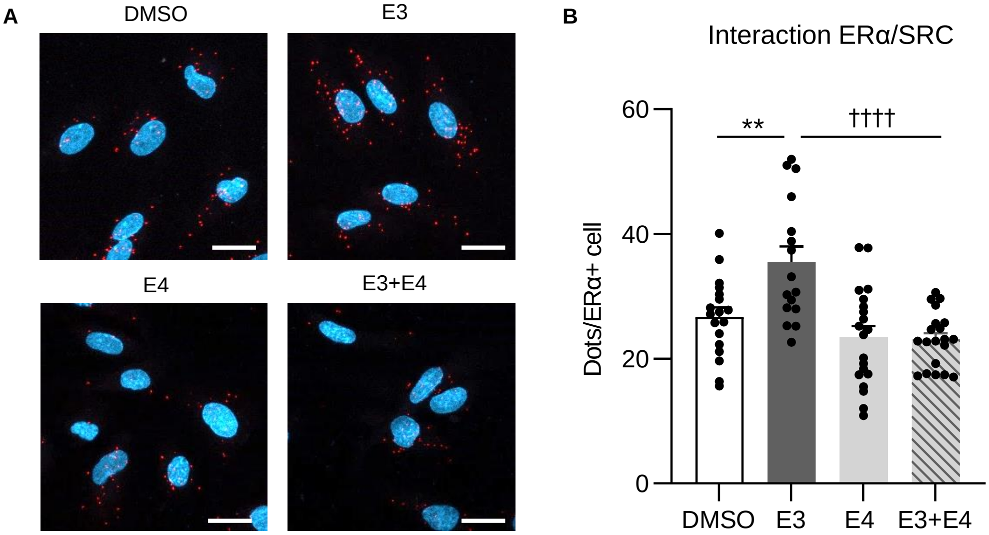

Supplemental Figure 5: Proximity Ligation Assay in ERα-TeloHAECs in response to E3. Estrogen-deprived ERα-TeloHAECs were incubated with DMSO, E3 10^{-6}M, E4 10^{-6}M or a combination of E3 and E4 for 5 min. Proximity ligation assay (PLA) for ERα/SRC interaction was performed. (**A**) Representative PLA images. The detected dimers are represented by red dots. Nuclei were counterstained with DAPI (blue) (Scale bar: 20µm). (**B**) Quantification of the number of dots per ERα-positive cell from one representative experiment. The experiment was replicated 2 times. Results are expressed as means ± SEM. To test the effect of the different treatments a 1-way ANOVA was performed. * indicates differences as compared to DMSO (***P*<0.01). † indicates differences between E3 and E3+E4 (††††*P*<0.0001).

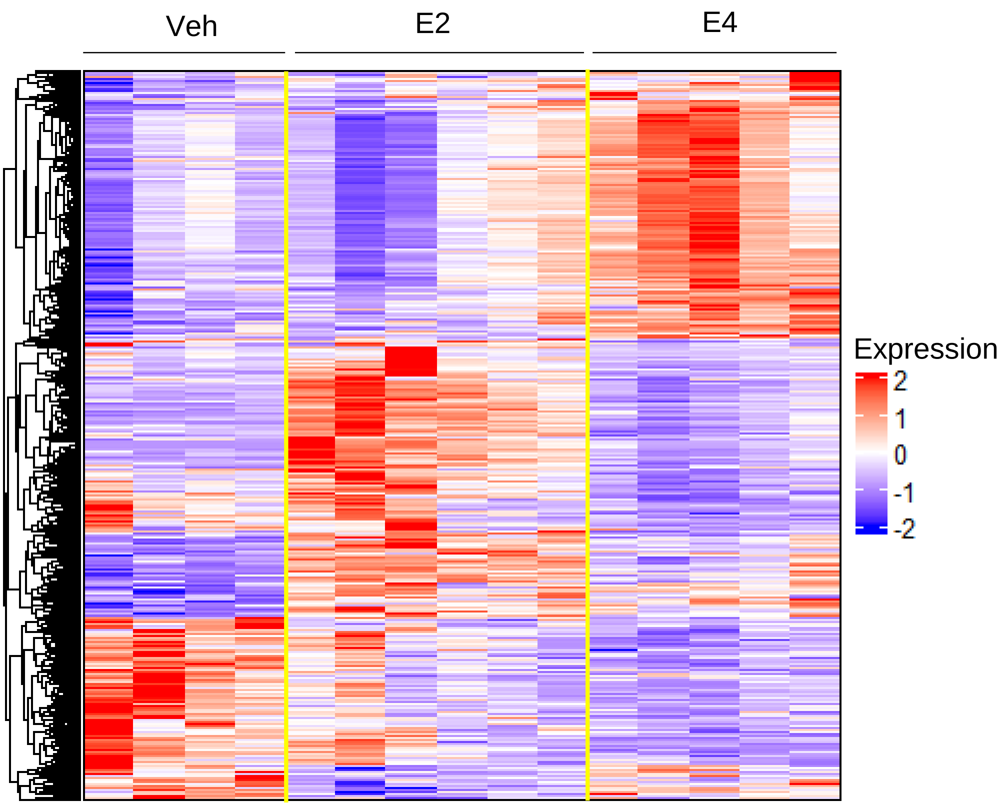

Supplemental Figure 6: Heatmap illustrating the relative expression values of all genes significantly regulated following E2 and E4 treatment. Fold change >2 or <0.5 over control with a BH (Benjamini-Hochberg) corrected $P<0.05$. Hierarchical clustering (HCL) regroups each sample with its corresponding treatment group (n = 4–6 per group).

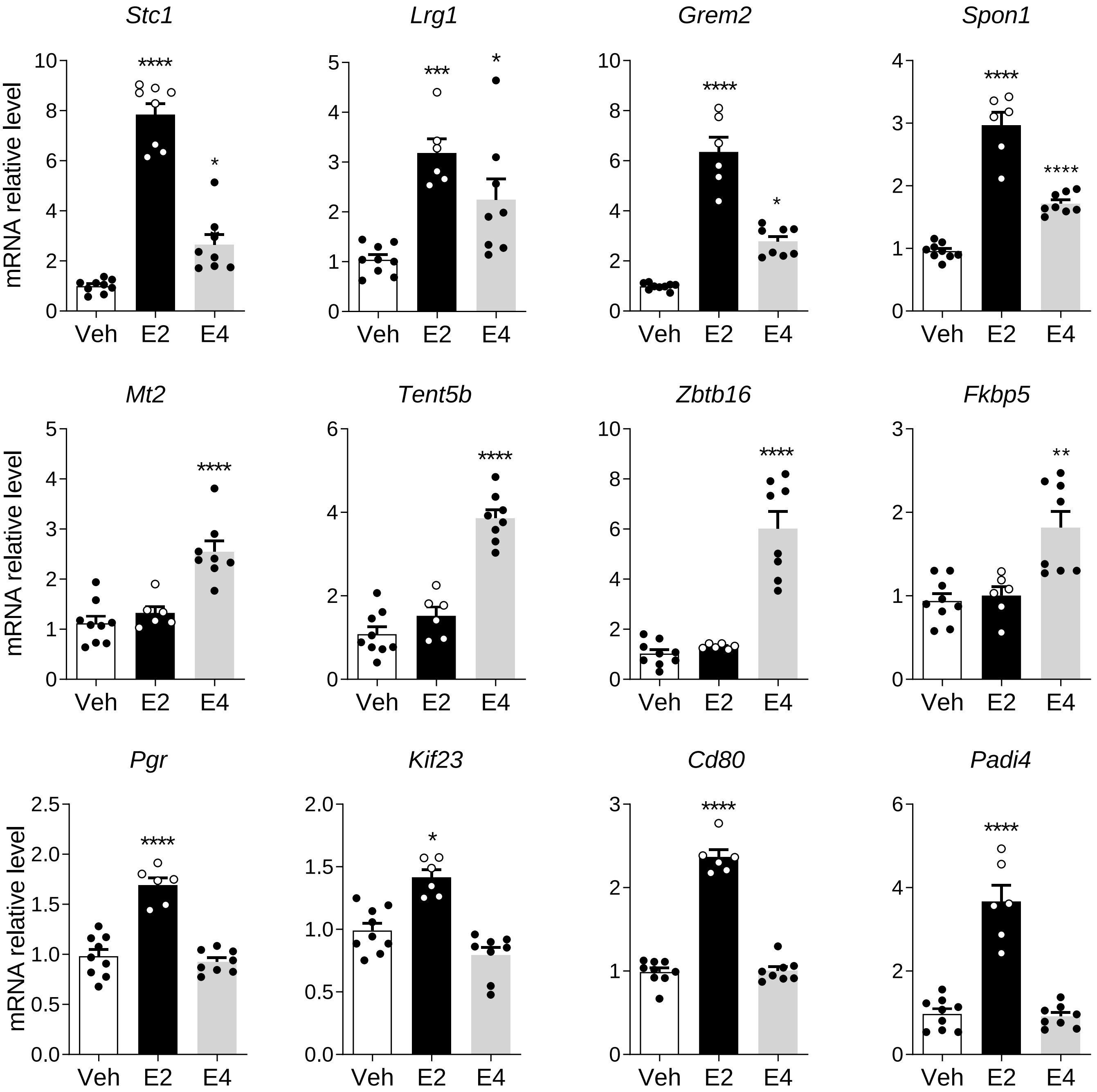

Supplemental Figure 7: RT-qPCR analysis of the mouse carotid artery in response to E2 and E4. RT-qPCR analysis of genes identified by RNA sequencing to be regulated by E2 and E4 (**top**), E4 only (**middle**) and E2 only (**bottom**) (n = 6–9 per group). Results are expressed as means ± SEM. One-way ANOVA followed by Bonferroni post-test was conducted. For data that failed normality testing, a Kruskal-Wallis with Dunn post-test was performed. * indicates differences as compared to Veh group (*P<0.05, **P<0.01, ***P<0.001, ****P<0.0001).

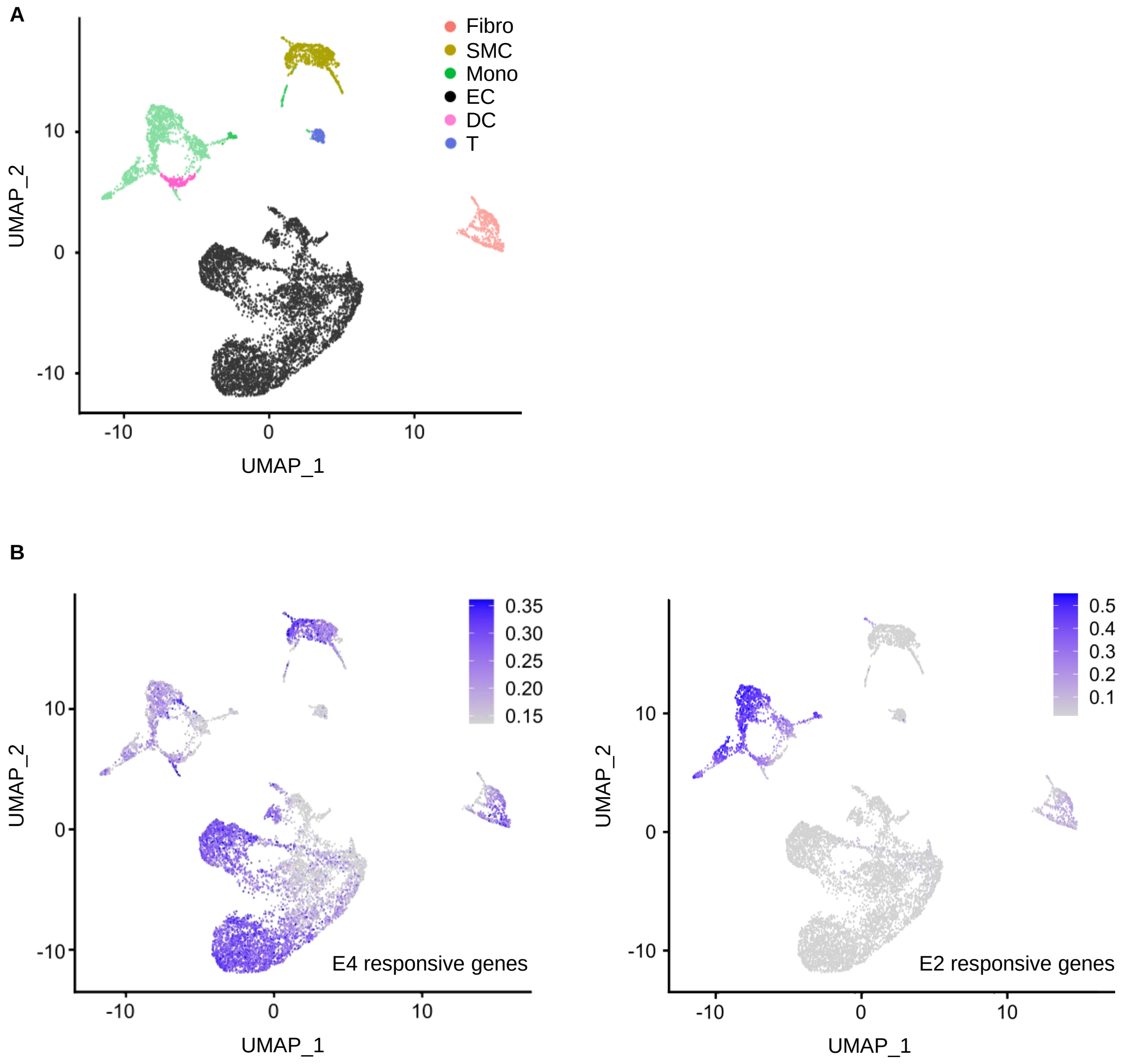

Supplemental Figure 8: Single-cell RNA-sequencing expression in ligated carotid arteries of E4- and E2-regulated genes. (**A**) Uniform Manifold Approximation and Projection (UMAP) of single-cell RNA-sequencing data from ligated carotid arteries of wild-type mice, organized by cell cluster. Fibro: fibroblast, SMC: smooth muscle cell, Mono: monocyte, EC: endothelial cell, DC: dentritic cell, T: T cell. (**B**) Feature plots of E4-regulated genes (**left**) and E2-regulated genes (**right**) identified by RNA-sequencing.

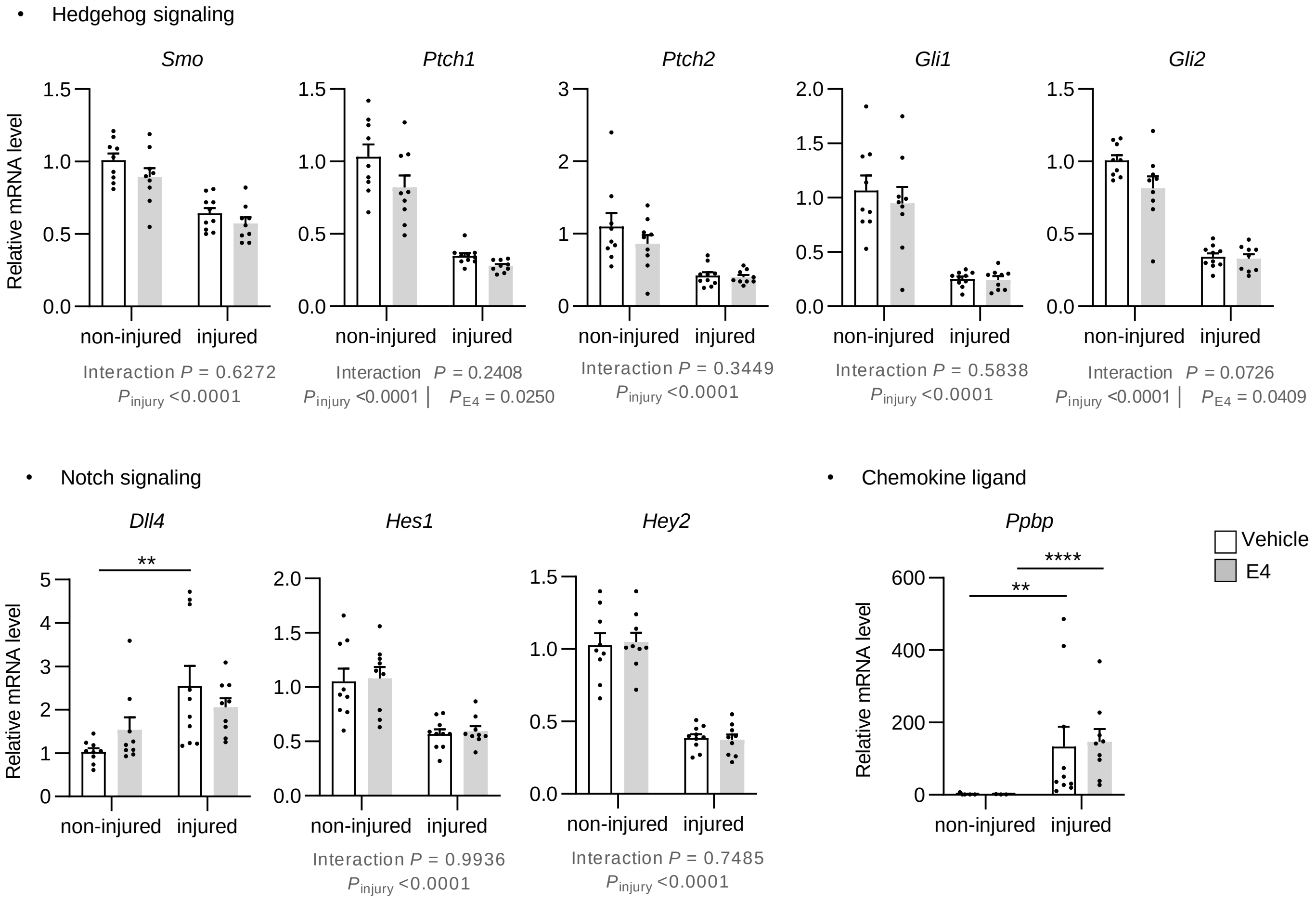

Supplemental Figure 9: Gene expression related to Hedgehog, Notch pathways and *Ppbp* in injured carotid arteries from mice treated by E4 *versus* Vehicle. Gene expression was analysed in non-injured and injured carotid arteries 24h after that endovascular injury was performed in Veh- and E4-treated female mice (*n* = 9–10 per group). Results are expressed as means ± SEM. To test the effect of injury and treatment a 2-way ANOVA was conducted. For data that failed normality testing, a Kruskal-Wallis with Dunn post-test was performed. (***P*<0.01, *****P*<0.0001).

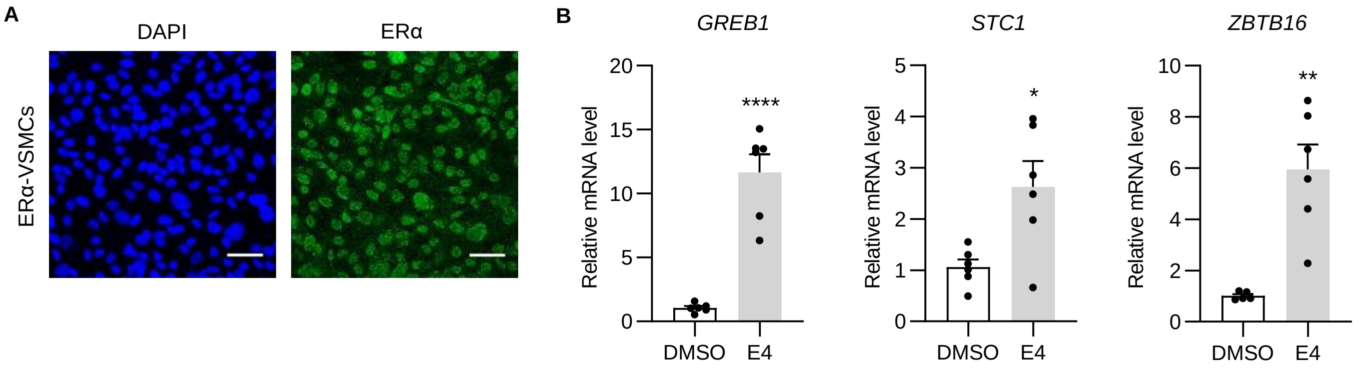

Supplemental Figure 10: Vascular smooth muscle cells expressing ERα respond to E4 treatment in vitro. Stable transduced vascular SMCs expressing full-length ERα (ERα-VSMCs) were deprived for 24h and then pretreated with DMSO or E4 10^{-6}M for 48h. (**A**) Representative ERα (green) and DAPI (blue) staining on ERα-VSMCs (scale bar: 50μm). Results were reproduced 2 times. (**B**) RT-qPCR analysis of E4 responsive genes in ERα-VSMCs at t48h (*n* = 5–6 per group from 2 independent experiments). Results are expressed as means ± SEM. To test the effect of E4 treatment, a Student t-test was performed (*P<0.05, **P<0.01, ****P<0.0001).

SUPPLEMENTAL TABLES

	[E2]	[E3]	[E4]
	(ng/mL)		
Veh pellet	< 0.010	< 0.010	< 0.050
E2 pellet	0.17 ± 0.02	< 0.010	< 0.050
E3 pellet	< 0.010	2.65 ± 0.19	< 0.050
E4 pellet	< 0.010	< 0.010	4.78 ± 0.57

Supplemental Table 1: Plasmatic E2, E3 and E4 measurements by GC-MS/MS. Four-week-old female mice were ovariectomized and implanted with either Veh, E2, E3 or E4 pellets. Plasma concentrations of E2, E3 and E4 were measured 2 weeks later. Results are expressed as means ± SEM (*n* = 7-11 per group).

Figure	Genotype	Treatment	Uterine weight (mg)	Statistics
2B	*C57BL/6J*	Veh	7.5 ± 1.1	*n* = 5-9 per group<br>One-way ANOVA<br>*P* < 0.0001
		E2	87.6 ± 6.6****	
		E3	77.4 ± 7.6****	
		E4	95.0 ± 8.7****	
		E2+E3	110.4 ± 6.3****	
		E2+E4	80.4 ± 8.6****	
		E3+E4	78.4 ± 2.4****	
3B	*αSMAcreER*^{T2} – *ERα*$^{lox/lox}$	Veh	11.0 ± 0.8	*n* = 5-6 per group<br>Two-way ANOVA<br>Interaction *P* = 0.9768<br>P_{E4} <0.0001
		E4	71.2 ± 4.1	
	αSMAcreER^{T2} + *ERα*$^{lox/lox}$	Veh	12.4 ± 0.5	
		E4	72.3 ± 7.4	
3C	*αSMAcreER*^{T2} – *ERα*$^{lox/lox}$	Veh	11.8 ± 0.8	*n* = 5-7 per group<br>Two-way ANOVA<br>Interaction *P* = 0.1748<br>P_{E3} <0.0001
		E3	95.4 ± 8.1	
	αSMAcreER^{T2} + *ERα*$^{lox/lox}$	Veh	12.8 ± 0.4	
		E3	79.3 ± 7.3	
4A	*WT-ERα*	Veh	7.6 ± 1.7	*n* = 6-7 per group<br>Two-way ANOVA<br>Interaction *P* = 0.2600<br>P_{E4} = 0.0004
		E4	80.4 ± 23.9	
	C451A-ERα	Veh	6.8 ± 1.5	
		E4	47.9 ± 11.1	
4B	*WT-ERα*	Veh	6.2 ± 9.5	*n* = 9-12 per group<br>Two-way ANOVA<br>Interaction *P* = 0.0576<br>P_{E4} <0.0001
		E4	110.8 ± 7.2	
	R264A-ERα	Veh	5.6 ± 0.4	
		E4	131.2 ± 8.4	
5F	*C57BL/6J*	Intact +Veh	83.4 ± 13.6	*n* = 5-6 per group<br>Student *t*-test
		Intact +E4	212.0 ± 7.0***	

Supplemental Table 2: Uterine weights of mice submitted to carotid artery injury. Results are expressed as means ± SEM. *** *P*<0.001, **** *P*<0.0001 vs Veh.

Gene Symbol	NCBI Reference Sequence	Species	Forward Sequence (5'-3')	Reverse Sequence (5'-3')
Tpt1	NM_009429.3	*Mus musculus*	TTGGATCTATCACCTGTCAACCA	TTTGTCCTAAAGTCCTGGTGTTGT
Esr1	NM_007956.5	*Mus musculus*	CTCCCGCCTTCTACAGGTCTAA	GACAGTCTCTCTCGGCCATTCT
Stc1	NM_009285.3	*Mus musculus*	GCACGAGGCGGAACAAAATGA	GTTGAGGCAGCGAACCACTTC
Lrg1	NM_029796.2	*Mus musculus*	CTCTCCACTCGCCACAACTCT	CAGTCAGCCTAGGAGCCGTTT
Grem2	NM_011825.1	*Mus musculus*	TCGTCATTGCAGGATGTTCTGG	AGGCCGGTTCTTCCGTGTTTC
Spon1	NM_145584.2	*Mus musculus*	TGTGTGATTCTGAAGGCCAGC	GTCCGTCACCCCATCAAGTGT
Mt2	NM_008630.2	*Mus musculus*	GCAAAGAGGCTTCCGACAAGTG	TGTGGAGAACGAGTCAGGGTTG
Tent5b	NM_175307.6	*Mus musculus*	AAAGAGCCGATCCCCATTCAC	AGTCCGTGTTCTTCCAAGCTG
Zbtb16	NM_001033324.3 and NM_001364543.1	*Mus musculus*	AGTTCAGCCTCAAGCACCAGT	GCACCGTTGTGTGTTCTCAGG
Fkbp5	NM_010220.4	*Mus musculus*	ATCAAACGGAAAGGCGAGGGA	TCTCTGCATCTTCACCAGGGC
Pgr	NM_008829.2	*Mus musculus*	AAACTGCCCAGCATGTCGTCT	AAACTGCCCAGCATGTCGTCT
Kif23	NM_024245.4	*Mus musculus*	AACTAGCCTCCGATGGGGAGA	GGTGGACGATCTTCGTTTCCG
Cd80	NM_001359898.1 and NM_009855.2	*Mus musculus*	ATACGACTCGCAACCACACCA	GGTCTTCTGGGGGTTTTTCCCA
Padi4	NM_011061.2	*Mus musculus*	AGGGTTTTCGGCTGCTGCTGTC	GCTCTCCACATAGGCATTCTGGTC
Smo	NM_176996.4	*Mus musculus*	TGGCCTGGTGCTTATTGTGGG	TCTTGCTGGCTGCCTTCTCACT
Ptch1	NM_008957.3	*Mus musculus*	TTCGCTCTGGAGCAGATTTCC	CACAACCAAAAACTTGCCGCAG
Ptch2	NM_008958.3	*Mus musculus*	AGTGCCATCCCCGTGGTAATC	GCAAAGGTCTGTTCCAGAGCG
Gli1	NM_010296.2	*Mus musculus*	CGACGGAGGTCTCTTTGTCCG	GGAAGGATGAGGGGACCTGGAGTT
Gli2	NM_001081125.1	*Mus musculus*	GCCCCACTCCAGCCAAGTT	TTTGGTGGCGGACCCGAG
Dll4	NM_019454.3	*Mus musculus*	CCCTTCAATTTCACCTGGCCG	TACCCACAGCAAGAGAGCCTT
Hes1	NM_008235.2	*Mus musculus*	GAGAAGAGGCGAAGGGCAAGA	CTTGGAATGCCGGGAGCTATCTTT
Hey2	NM_013904.1	*Mus musculus*	GAAGATGCTCCAGGCTACAGGG	TGAGATGAGAGACAAGGCGCA
Ppbp	NM_023785.3	*Mus musculus*	GCCTGCCCACTTCATAACCT	ATTCGTACATCTGCAGCGCA
Cxcl10	NM_021274.2	*Mus musculus*	TCCGGATTCAGACACCTCTTCTC	TGTCCGCATGTTGAGATCATTGC
TBP	NM_003194.5	*Homo sapiens*	TAAGAGAGCCACGAACCACGG	GCTGCCAGTCTGGACTGTTCT
GREB1	NM_014668.4	*Homo sapiens*	TTCCCCGAAGTGCCAACAACT	ACTTAGCTCTGTTCCCACCACC
STC1	NM_003155.3	*Homo sapiens*	GACTCTGTGAGCCCCAGGAAA	AGCACTGTTGAGGCAACGAAC
ZBTB16	NM_006006.6	*Homo sapiens*	GTCTCCATGGACTTCAGCAC	TACGTCTTCATCCCACTGTG
CXCL10	NM_001565.4	*Homo sapiens*	GAACCTCCAGTCTCAGCACCA	AATGCTGATGCAGGTACAGCG

Supplemental Table 3: List of primers for RT-qPCR.

Steroids (Molecular weight)	Derivatized steroids (molecular weight)	Retention time (min.)	Transition (m/z→m/z)	Collision energy (eV)
Estetrol (304)	Estetrol-3,15,16,l7-HFB_4 (1088)	17.90	447→233	8
Estriol (288)	Estriol-3,16,l7-HFB_3 (876)	18.49	876→235	16
17b-Estradiol (272)	17b-Estradiol-3,l7-HFB_2 (664)	19.25	664→237	10
		Internal standards		
$^{2}H_4$-Estetrol (308)	$^{2}H_4$-Estetrol-3,15,16,l7-HFB_4 (1092)	17.88	451→237	8
$^{13}C_3$-Estriol (291)	$^{13}C_3$-Estriol-3,16,l7-HFB_3 (896)	18.49	879→238	16
$^{2}H_5$-17b-Estradiol (277)	$^{2}H_5$-17b-Estradiol-3,l7-HFB_2 (669)	19.21	669→242	10

Supplemental Table 4: GC-MS/MS parameters for steroids identification and quantification in multiple reaction monitoring detection mode. The name of steroids and derivatized steroids are indicated with their respective molecular weight. The retention time and the transition used for quantification are indicated. A transition is defined by the selection of a parent ion with the first mass spectrometer that is dissociated in a collision cell with Argon at optimal collision energy. The generated fragment ion is analyzed with the second mass spectrometer and detected.

SUPPLEMENTAL METHODS

En Face Immunostaining

Injured carotid arteries were dissected, fixed for 20 min in 4% paraformaldehyde, opened longitudinally, and incubated for 1 hour with PBS containing 0.1% TritonX100, 2% BSA and 1% FBS for permeabilisation and blocking. Carotid were immunostained with anti-VE-cadherin rat monoclonal primary antibody (1:100; BD Pharmingen; 555289) overnight at 4°C. Then, carotids were incubated with AlexaFluor®488-conjugated anti-rat secondary antibody (1:200; Jackson ImmunoResearch; 712-545-150) for 2 hours at room temperature. Nuclei were stained with DAPI (1 µg/mL) and carotids were mounted with Dako Mounting Medium (Agilent Technologies #S3023). Microscopy imaging was performed with a Zeiss LSM780 confocal microscope.

Real Time qPCR Analysis

RNA isolation was performed as described for RNA sequencing. 500 ng of total RNA were reverse transcribed 10 min at 25°C followed by 2 h at 37°C using the High Capacity cDNA reverse transcriptase kit (Applied Biosystems). qPCR were performed using SsoFast EvaGreen Supermix (Bio-Rad) on a StepOne instrument (Applied Biosystems). Gene expression was quantified using the comparative Ct (threshold cycle) method. Tumor protein, translationally-controlled 1 (*Tpt1)* (for mouse arteries) and TATA-box binding protein (*TBP)* (for human VSMCs) were used as housekeeping gene to normalize the mRNA levels. The list of primers used is provided in the **Supplemental Table 3**, primers efficiency were evaluated using LinReg software (95% < efficiency < 105%).

Steroid measurements by gas chromatography tandem mass spectrometry (GC-MS/MS)

Serum from 1) ovariectomized mice treated by either vehicle or E2, E3 or E4 pellets; and 2) gonad-intact female mice treated by either vehicle or E4 pellets were used for steroid measurements. Steroids were identified and quantified simultaneously in serum by gas chromatography tandem mass spectrometry (GC-MS/MS) as previously described (1). Steroids were extracted from serum (62 – 200 µl) with 2 ml MeOH. The following internal standards were introduced into the extracts for steroid quantification: 1 ng of 2H5-E2 (Cluzeau Info Labo, Sainte-Foy-La-Grande, France) for the analysis of E2, 1 ng of 13C3-E3 (Isosciences, PA, USA) for the analysis of E3 and 5 ng of 2H4-E4 (Clinisciences, Nanterre, France) for the analysis of E4.

Samples were purified and fractionated by solid-phase extraction with the recycling procedure (2). Briefly, the extracts were dissolved in 1 ml MeOH and applied to the C18 cartridge (500 mg, 6 ml, International Sorbent Technology, IST), followed by 5 ml of MeOH/H2O (85/15). The flow-through, containing the unconjugated steroids, was collected and dried. After a previous re-conditioning of the same cartridge with 5 ml MeOH/H2O (20/80), the dried samples were dissolved in MeOH/H2O (2/8) and re-applied. The cartridge was then washed with 5 ml H2O and 5 ml MeOH/H2O (2/8) and unconjugated steroids were eluted with 5 ml MeOH/H2O (9/1).

The unconjugated steroids-containing fraction was then filtered and further purified by HPLC. The HPLC system is composed of a WPS-3000SL analytical autosampler and a LPG-3400SD quaternary pump gradient coupled with a SR-3000 fraction collector (Thermoscientific, USA). The HPLC separation was achieved with a Lichrosorb Diol column (25 cm, 4.6 mm, 5 µm) in a thermostatic block at 30° C. The column was equilibrated in a solvent system of 90% heptane and 10% of a mixture composed of heptane/isopropanol (85/15). Elution was performed at a flow-rate of 1 ml/min, first 90% heptane and 10% of heptane/isopropanol (85/15) for 15 min, then with a linear gradient to 100% of acetone in 2 min. This mobile phase was kept constant for 13 min. The fraction containing E2, E3 and E4 and their respective internal standards were collected between 15 and 29 min.

This fraction was derivatized with 25 µl heptafluorobutyric anhydride (HFBA) and 25 µl anhydrous acetone for 1h at 80°C. Samples were dried under a stream of N2 and resuspended in heptane for GC-MS/MS analysis.

GC-MS/MS analysis of the extracts was performed using an AI 1310 autosampler, a Trace 1310 gas chromatograph (GC), and a TSQ 8000 tandem mass spectrometer (MS/MS) (Thermo Fisher Scientific San Jose, CA) using Argon as collision gas. Injection was performed in the splitless mode at 280°C (1 min of splitless time) and the temperature of the gas chromatograph oven was initially maintained at 80°C for 1 min and ramped between 50 to 350°C at 10°C/min. The helium carrier gas flow was maintained constant at 1 ml/min during the analysis. The transfer line and ionization chamber temperatures were 330°C and 200°C, respectively. Electron impact ionization was used with ionization energy of 70 eV and an emission current of 50 µA for mass spectrometry. Mass spectrometry acquisitions were performed in Multiple Reaction Monitoring (MRM) mode. GC/MS/MS signals were evaluated using a computer workstation by means of the software Excalibur®, release 3.0 (Thermoscientific, USA). Identification of steroids was supported by their retention time and according three transitions. Quantification was performed according to the transition giving the more abundant product ion (**Supplemental Table 4**) with a previously established calibration curve.

The analytical protocol has been validated by using extracts of 200 µl from a pool of male mice serum. The evaluation included the limit of detection, linearity, accuracy, intra- and inter-assay precisions. The limit of detection, determined as the lowest amount of compounds that can be measured with a signal-to-noise ratio greater than 3, was 1, 2 and 5 pg/ml for E2, E3 and E4, respectively. The linearity was assessed by analysing increasing amounts of mice serum extracts (50, 100 and 200 µl) in triplicate. The linearity was satisfactory for all the steroids with a coefficient of correlation ranging from 0.992 to 0.999. The accuracy of the assay was evaluated by determining the analytical recovery, which was defined as C/(C0+S)x100(%). C is the concentration of the steroid in the spiked serum extract (100 µl), C0 is the concentration of a steroid in the unspiked serum extract (100 µl) and S is the spiked concentration. Accuracy of E2, E3 and E4 was 97.1, 95.6 and 104.3 %, respectively. The precision of the intra and inter-assays, evaluated by analysing 5 replicates of 200 µl of serum extracts on 1 day and over 4 days, respectively. The intra-assay precision was 4.2, 5.3 and 6.1 % and the inter-assay precision was 5.8, 5.9 and 8.4 % for E2 and E4, respectively.

Immunohistochemistry on uterine sections

Paraffin-embedded transverse sections (4-µm) from formalin-fixed uteri were dewaxed in toluene and rehydrated through acetone bath to deionized water. Antigen retrieval was performed in 10 mM citrate buffer (pH 6.0) for 30 minutes in a water bath at 95°C. Cooled sections were then incubated in peroxidase blocking solution (DAKO) to quench endogenous peroxidase activity. To block nonspecific binding, sections were incubated in normal goat serum (DAKO) for 20 minutes at room temperature. Sections were incubated 50 minutes at room temperature with rabbit anti-ERα primary antibody (1/300; Santa Cruz; sc542). The secondary antibody, biotinylated goat anti-rabbit Igs (ready-to-use; Microm; F/TP-060-BN), was applied for 25 minutes at room temperature followed by an horseradish peroxidase-streptavidin solution (1/500; DAKO) for 25 minutes. Peroxidase activity was revealed by 3,3′-diaminobenzidine tetrahydrochloride substrate (DAKO). Finally, sections were counterstained with Harris hematoxylin, dehydrated, and coverslipped.

References:

1. Zhu X, et al. A Role of Endogenous Progesterone in Stroke Cerebroprotection Revealed by the Neural-Specific Deletion of Its Intracellular Receptors. J Neurosci 2017;37(45):10998–11020.
2. Liere P, et al. Novel lipoidal derivatives of pregnenolone and dehydroepiandrosterone and absence of their sulfated counterparts in rodent brain. J Lipid Res 2004;45(12):2287–2302.
